# Supplementary material for: Distinct Cdk9-phosphatase switches act at the beginning and end of elongation by RNA polymerase II
Source: Nat Commun. 2020 Aug 28;11:4338. doi: 10.1038/s41467-020-18173-6 (PMC7455706; doi:10.1038/s41467-020-18173-6)
Supplement: Supplementary file 1 — Supplementary Information [file 41467_2020_18173_MOESM1_ESM.pdf]

Distinct Cdk9-phosphatase switches act at the beginning and end of elongation by RNA polymerase II

Pabitra K. Parua, Sampada Kalan, Bradley Benjamin, Miriam Sansó and Robert P. Fisher*

Department of Oncological Sciences, Icahn School of Medicine at Mount Sinai, New York, NY, 10029-6574, USA

*Corresponding author: Robert.fisher@mssm.edu

Running title: Cdk9-phosphatase circuits regulate transcription elongation

Supplementary Figures and Legends

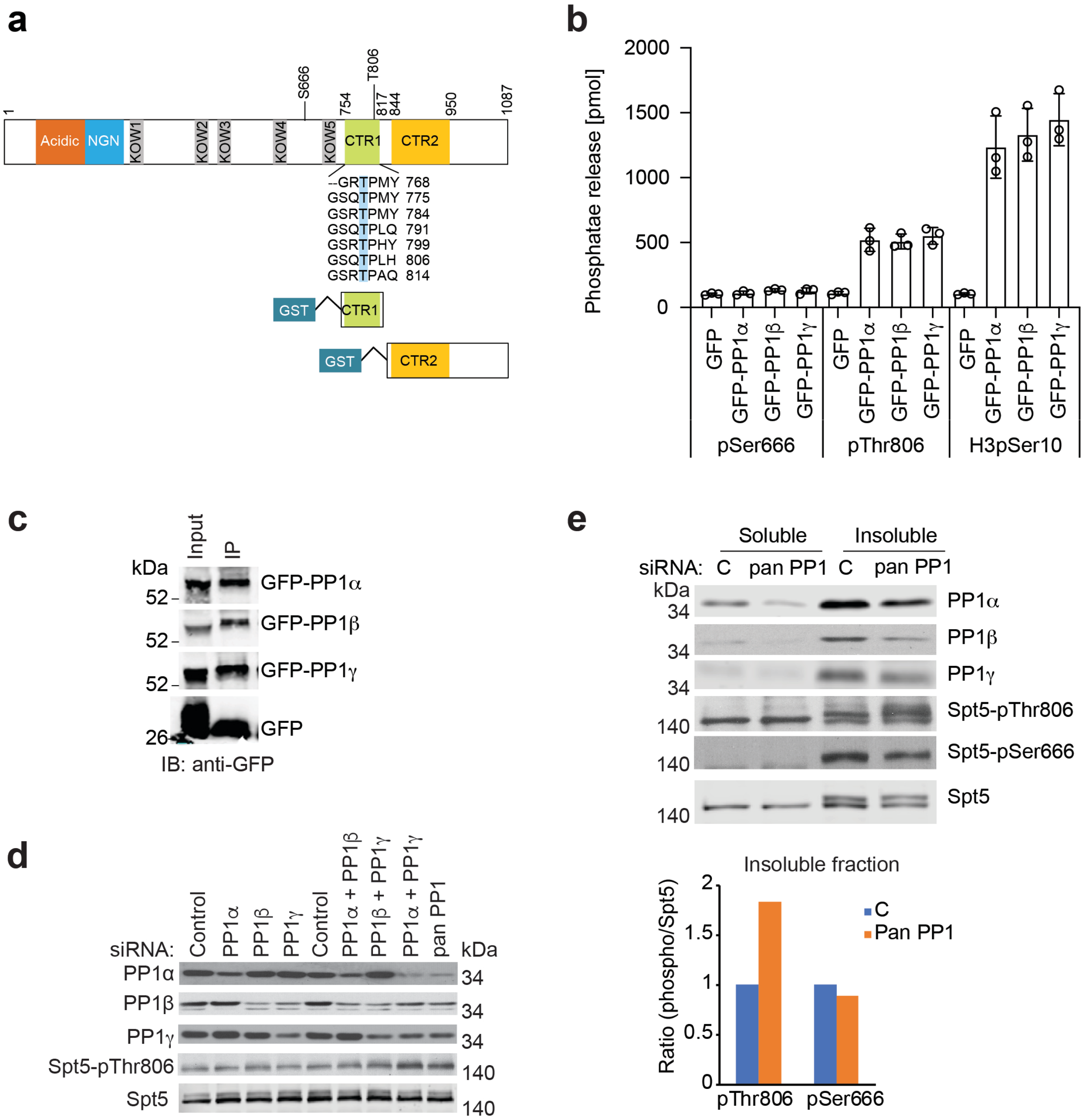

Supplementary Fig. 1 PP1 regulates Spt5 phosphorylation in human cells. a Schematic diagram of Spt5 protein. **b** Immunoprecipitated GFP-tagged PP1 isoforms (PP1α, PP1β and PP1γ) dephosphorylated pThr806-containing phosphopeptide and a control (H3pSer10), but not pSer666. Phosphate release was measured colorimetrically. Individual data points are shown in the plot; error bars indicate ± s.d. of three biological replicates (*n* = 3). **c** Anti-GFP immunoblot shows amounts of PP1 isoforms immunoprecipitated. **d** Immunoblot to measure depletion of PP1 isoforms (PP1α, PP1β and PP1γ) using isoform-specific siRNAs (singly or in combinations) and levels of Spt5, and Spt5-pThr806. **e** Immunoblot (top) of chromatin fraction (insoluble) indicates pThr806 was increased, whereas pSer666 was unchanged in cells depleted of all

three PP1 isoforms with an siRNA cocktail. Signals were scanned and quantified (bottom) with ImageJ software (*n* =1 biologically independent experiment). Source data are provided as a Source Data file.

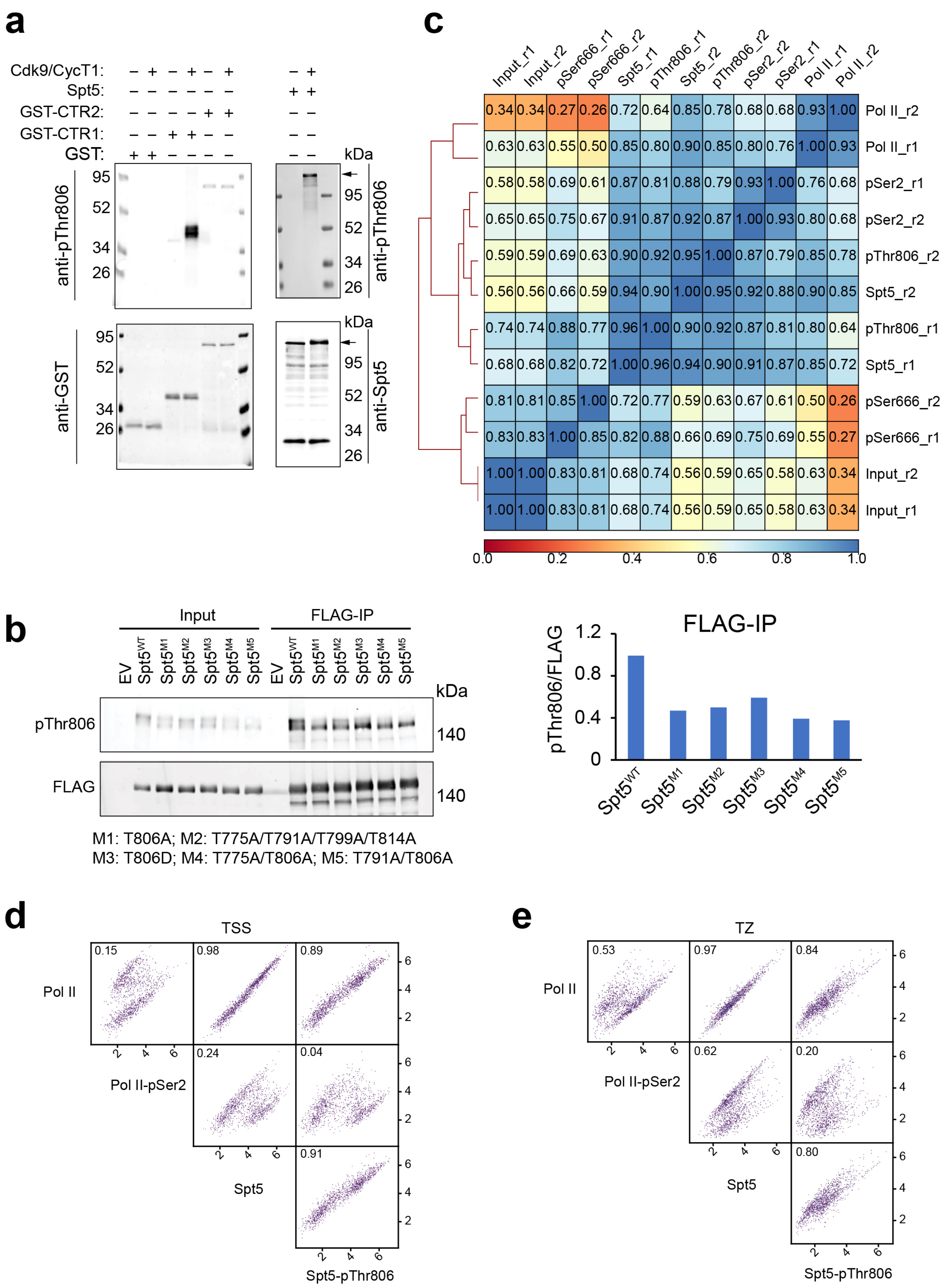
a
Cdk9/CycT1:
Spt5:
GST-CTR2:
GST-CTR1:
GST:
kDa
95
52
34
26
anti-pThr806
anti-GST
anti-Spt5
c
Input_r1 Input_r2 pSer666_r1 pSer666_r2 Spt5_r1 pThr806_r1 Spt5_r2 pThr806_r2 pSer2_r2 pSer2_r1 Pol II_r1 Pol II_r2
0.34 0.34 0.27 0.26 0.72 0.64 0.85 0.78 0.68 0.68 0.93 1.00 Pol II_r2
0.63 0.63 0.55 0.50 0.85 0.80 0.90 0.85 0.80 0.76 1.00 0.93 Pol II_r1
0.58 0.58 0.69 0.61 0.87 0.81 0.88 0.79 0.93 1.00 0.76 0.68 pSer2_r1
0.65 0.65 0.75 0.67 0.91 0.87 0.92 0.87 1.00 0.93 0.80 0.68 pSer2_r2
0.59 0.59 0.69 0.63 0.90 0.92 0.95 1.00 0.87 0.79 0.85 0.78 pThr806_r2
0.56 0.56 0.66 0.59 0.94 0.90 1.00 0.95 0.92 0.88 0.90 0.85 Spt5_r2
0.74 0.74 0.88 0.77 0.96 1.00 0.90 0.92 0.87 0.81 0.80 0.64 pThr806_r1
0.68 0.68 0.82 0.72 1.00 0.96 0.94 0.90 0.91 0.87 0.85 0.72 Spt5_r1
0.81 0.81 0.85 1.00 0.72 0.77 0.59 0.63 0.67 0.61 0.50 0.26 pSer666_r2
0.83 0.83 1.00 0.85 0.82 0.88 0.66 0.69 0.75 0.69 0.55 0.27 pSer666_r1
1.00 1.00 0.83 0.81 0.68 0.74 0.56 0.59 0.65 0.58 0.63 0.34 Input_r2
1.00 1.00 0.83 0.81 0.68 0.74 0.56 0.59 0.65 0.58 0.63 0.34 Input_r1
0.0 0.2 0.4 0.6 0.8 1.0
b
Input
FLAG-IP
EV Spt5WT Spt5M1 Spt5M2 Spt5M3 Spt5M4 Spt5M5
kDa
pThr806
FLAG
140
M1: T806A; M2: T775A/T791A/T799A/T814A
M3: T806D; M4: T775A/T806A; M5: T791A/T806A
FLAG-IP
pThr806/FLAG
1.2
0.8
0.4
0
Spt5WT Spt5M1 Spt5M2 Spt5M3 Spt5M4 Spt5M5
d
TSS
Pol II
Pol II-pSer2
Spt5
Spt5-pThr806
0.15 0.98 0.89
0.24 0.04
0.91
e
TZ
Pol II
Pol II-pSer2
Spt5
Spt5-pThr806
0.53 0.97 0.84
0.62 0.20
0.80

Supplementary Fig. 2 ChIP-seq analysis of Spt5-pThr806 and Pol II-pSer2. a Immunoblot analysis shows that upon phosphorylation by Cdk9, anti-pThr806 antibody recognizes GST-CTR1 (amino acids 720-830) and full-length protein, but not GST-CTR2 (amino acids 844-1087); top (anti-pThr806), bottom left (anti-GST), and bottom right (anti-Spt5). **b** Ectopically expressed FLAG-tagged Spt5—wild-type (WT) and indicated CTR1-mutant variants (M1-M5)—was directly immunoblotted or immunoprecipitated with anti-FLAG (M2) antibody and probed with anti-pThr806 and anti-FLAG antibodies, as indicated (left). Signals were quantified using Image Studio software in Odyssey CLx Imager (right) (*n* =1 biologically independent experiment). **c** Correlation between ChIP-seq samples. Paired-end sequencing reads were mapped to human genome using Bowtie2. Average read coverages were calculated by feeding resulting BAM files into 'multiBamSummary' program (Galaxy Version 3.1.2.0.0). Values in boxes represent Pearson's correlation coefficients between corresponding samples (*n* = 2 biological replicates). **d** Scatterplots represent correlations between Pol II, Pol II-pSer2, Spt5 and Spt5-pThr806 downstream of the TSS (0 to +200 bp) for genes highly occupied by Pol II (*n* = 2,572). **e** Scatterplots represent correlations between Pol II, Pol II-pSer2, Spt5 and Spt5-pThr806 at termination zone (TZ; 0 to +2,500 bp of the CPS) for genes highly occupied by Pol II (*n* = 2,572). Source data are provided as a Source Data file.

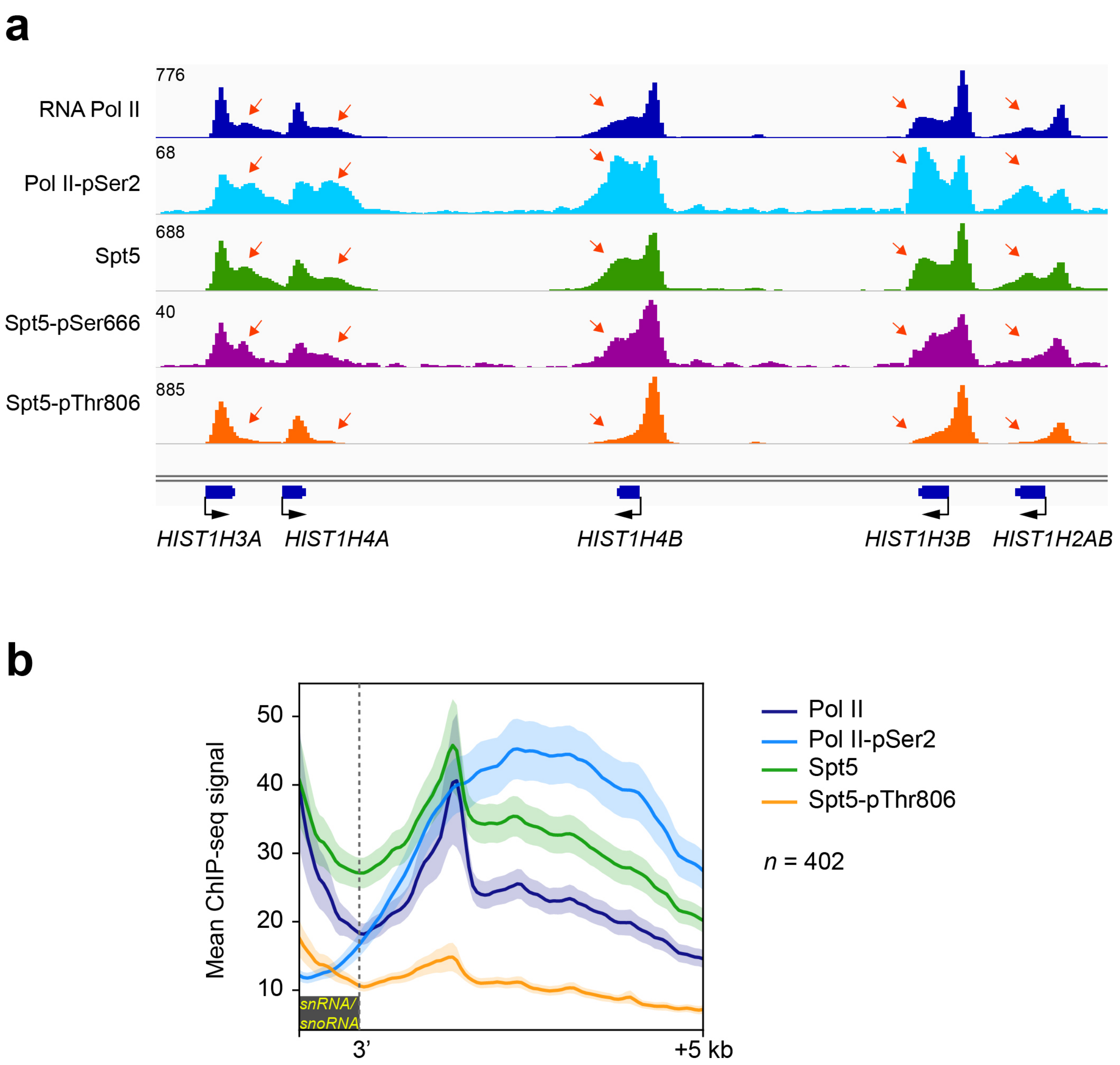

Supplementary Fig. 3 Spt5-CTR1 phosphorylation drops in termination zones of replication-dependent histone and noncoding RNA genes transcribed by Pol II. a Browser tracks of representative histone genes, with 3' regions indicated by red arrows, where ChIP-seq signals of Pol II, pSer2, Spt5 and pSer666, but not pThr806, accumulate. **b** Metagene analysis of Pol II, pSer2, Spt5 and pThr806 ChIP-seq signals on snRNA and snoRNA genes, with annotated 3' end indicated by dashed vertical line. The shaded area represents standard deviation (± s.d.) from the mean distribution (solid line).

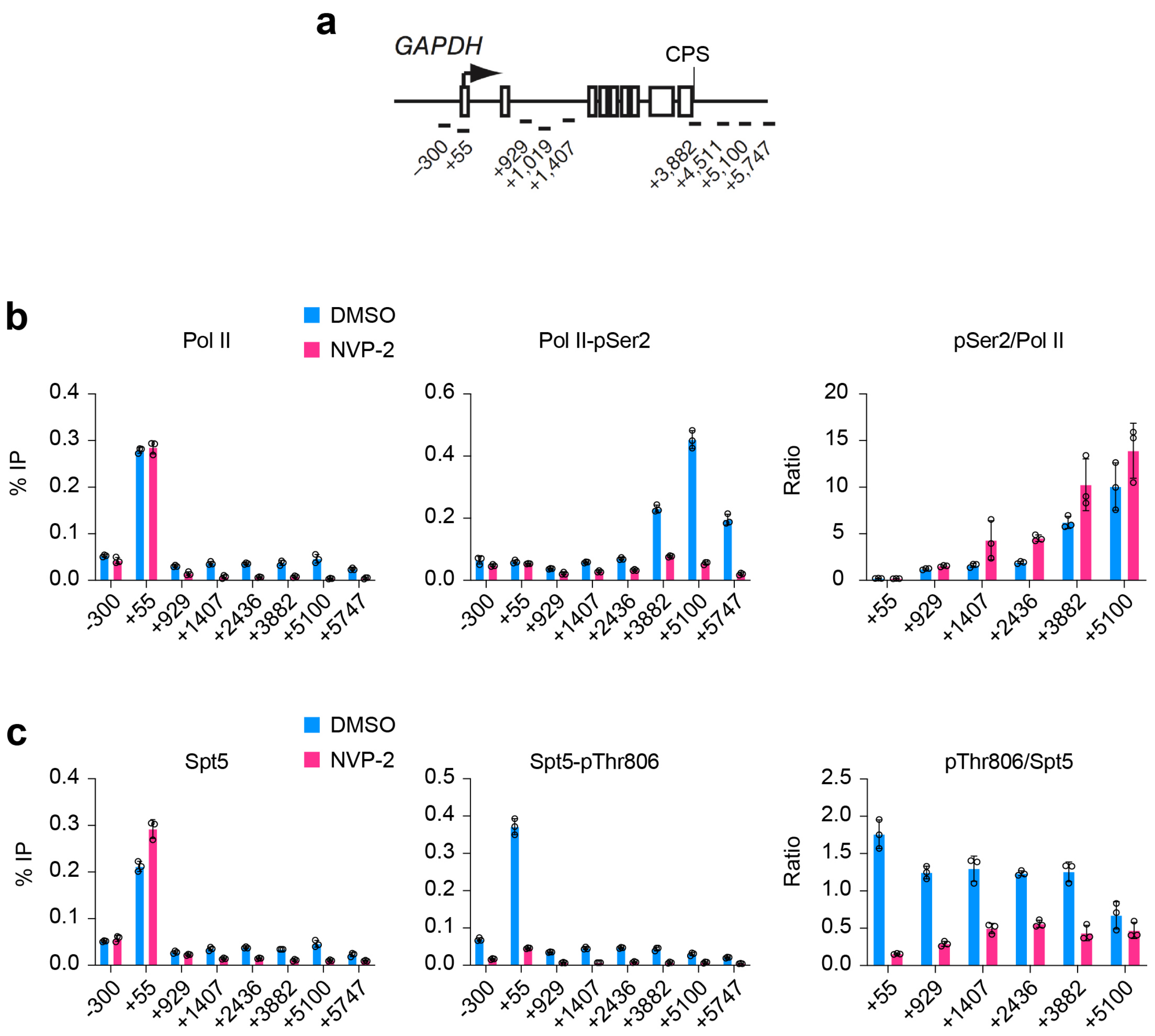

Supplementary Fig. 4 Cdk9 inhibition diminishes Spt5-pThr806 but increases Pol II CTD-pSer2 on *GAPDH*. a Schematic of the *GAPDH* gene, indicating positions of primer pairs used in ChIP-qPCR analysis. **b** ChIP-qPCR analysis of total Pol II and CTD-pSer2 in HCT116 cells treated with NVP-2 (250 nM) or mock treated (DMSO) for 1 hr. **c** ChIP-qPCR analysis of total Spt5 and Spt5-pThr806 in HCT116 cells treated with NVP-2 (250 nM) or mock treated (DMSO) for 1 hr. Individual data points are shown in the plots; error bars indicate ± s.d. of three biological replicates (**b** and **c**). Source data are provided as a Source Data file.

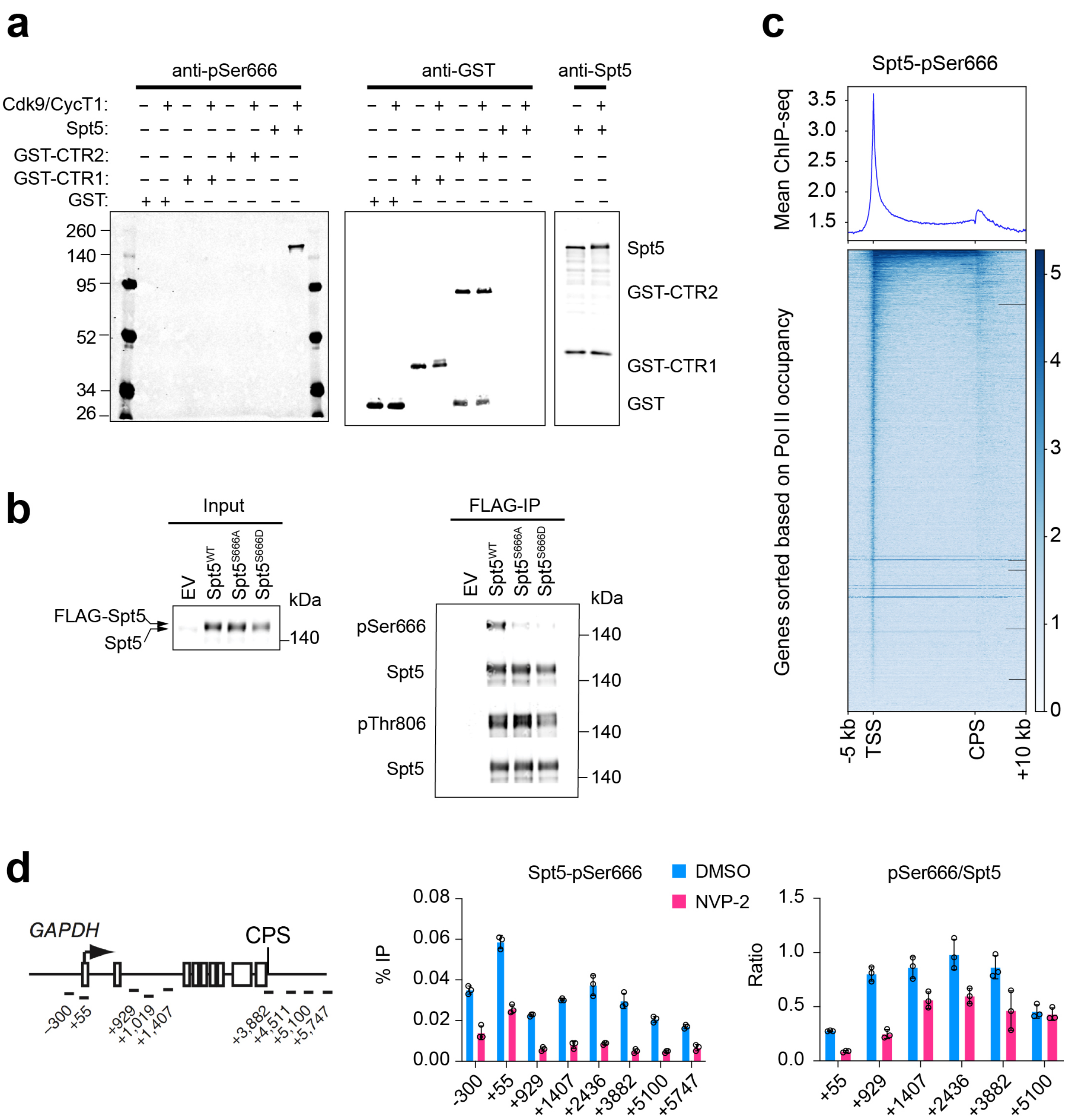

Supplementary Fig. 5 Distribution and phosphorylation of Spt5 and Pol II-Ser2 on chromatin. a Immunoblot analysis shows that upon phosphorylation by Cdk9, anti-pSer666 antibody recognizes full-length Spt5 protein, but not GST-CTR1 or GST-CTR2; blots were probed with anti-pSer666 (left), anti-GST (middle), and anti-Spt5 (right). **b** Ectopically expressed FLAG-tagged Spt5—wild-type (WT) and Ser666-mutant variants ($Spt5^{S666A}$ and $Spt5^{S666D}$)—was directly immunoblotted (top, "input") or immunoprecipitated with anti-FLAG antibody (bottom, "FLAG-IP") and probed with anti-pSer666, anti-Spt5, anti-pThr806 antibodies. **c** Metagene analysis and heat map (*n* = 20,130 genes) of ChIP-seq data for Spt5-pSer666 (*n* = 2 biological replicates). **d** ChIP-qPCR analysis of Spt5-pSer666 on *GAPDH* in HCT116 cells treated with NVP-2 (250 nM) or mock treated (DMSO) for 1 hr. Individual data points are shown in the plots;

error bars indicate ± s.d. of three biological replicates (**b** and **c**). Source data are provided as a Source Data file.

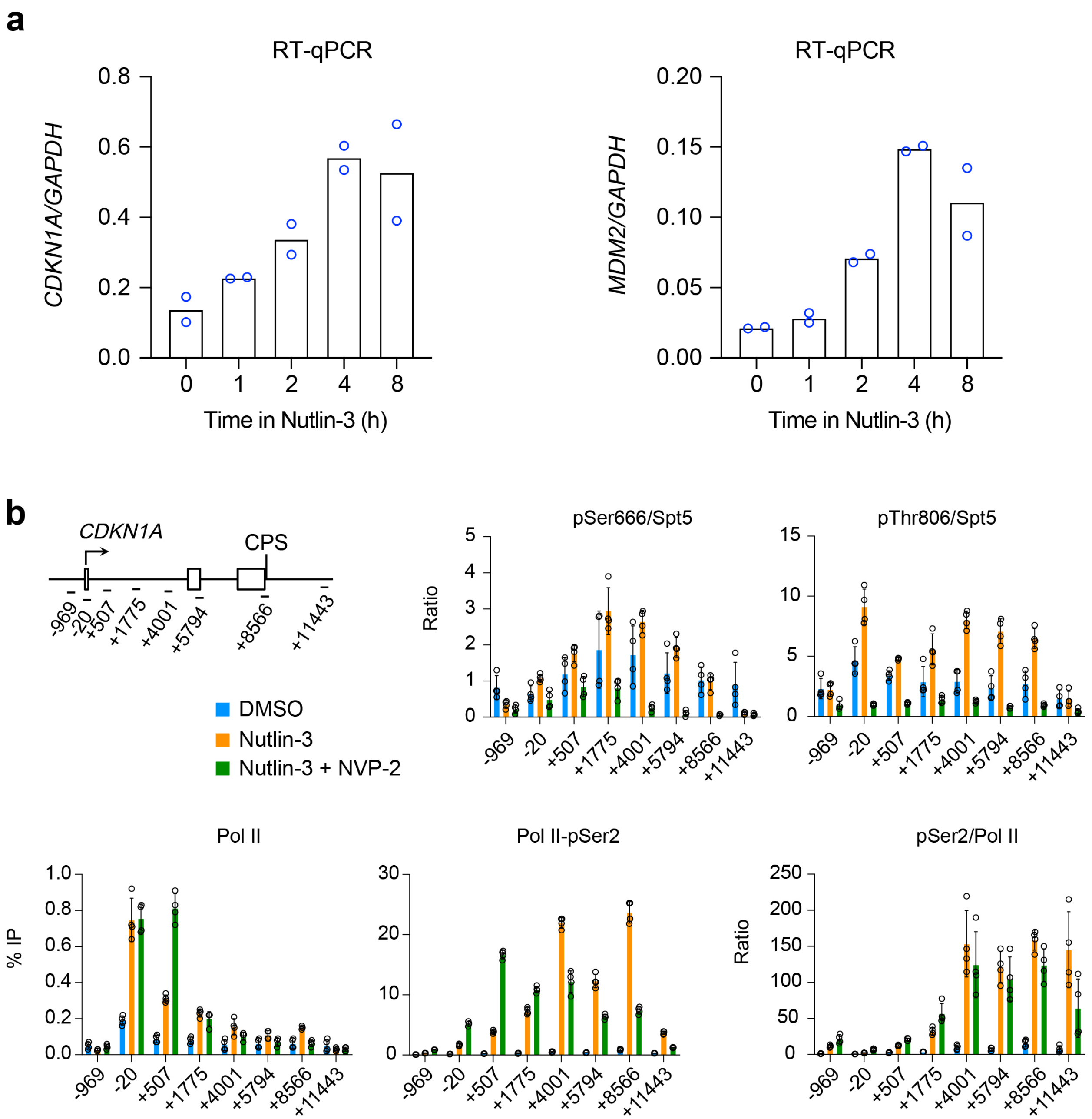

Supplementary Fig. 6 Spt5 phosphorylation in response to p53 activation in the absence or presence of a Cdk9 inhibitor. a Time-dependent induction of two p53-responsive genes, *CDKN1A* and *MDM2*, in response to treatment with nutlin-3 (5 μM), measured by RT-qPCR amplification relative to *GAPDH* control. Individual data points from two biological replicates (n = 2) are shown. **b** Schematic of *CDKN1A* gene, indicating positions of primer pairs used in ChIP-qPCR analysis, and ChIP-qPCR analysis of Spt5-pSer666, Spt5-pThr806, Pol II and Pol II-pSer2 in HCT116 cells mock treated (DMSO) or treated with nutlin-3 (5 μM) alone or together with NVP-2 (250 nM). Individual data points are shown in the plots; error bars indicate ± s.d. from four biological replicates (n = 4). Source data are provided as a Source Data file.

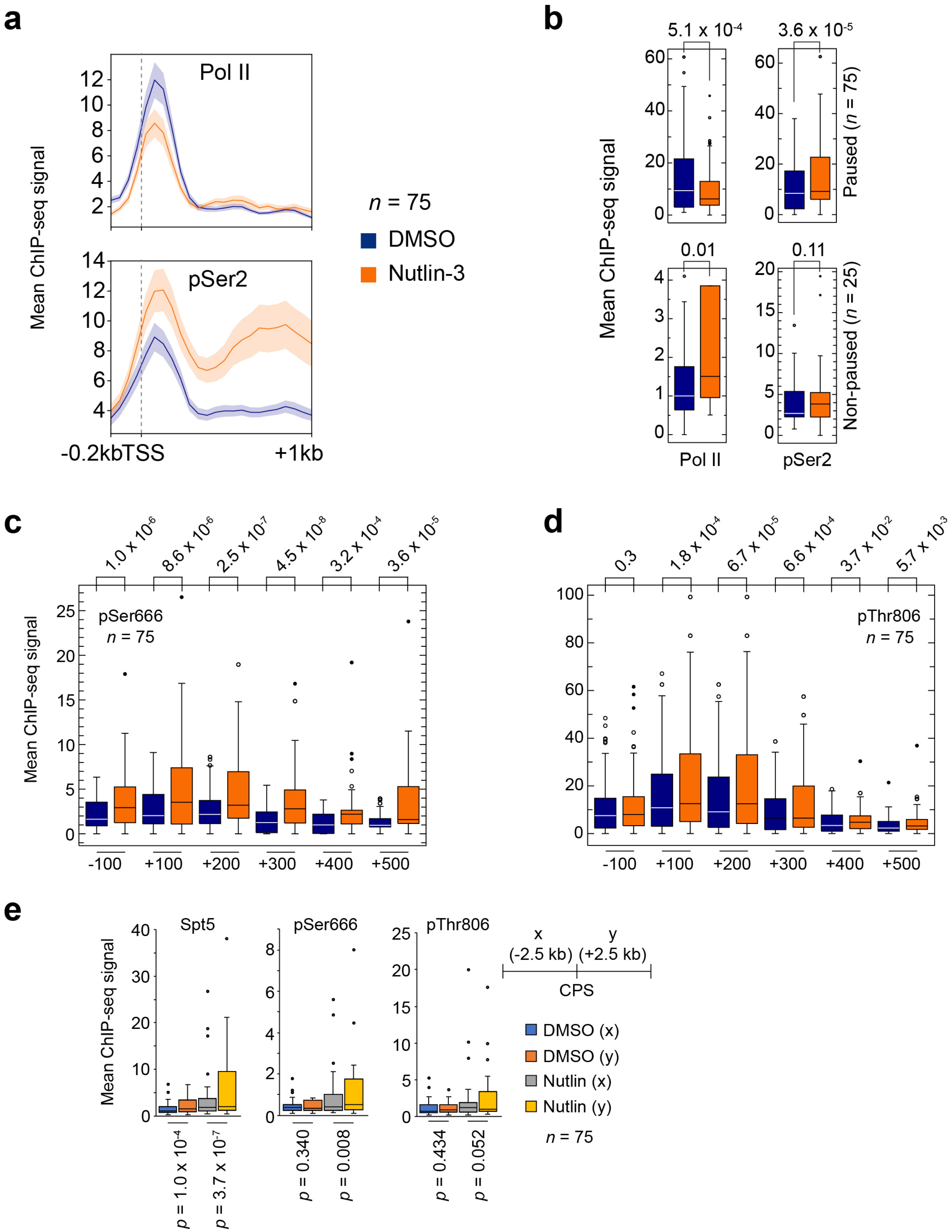

Supplementary Fig. 7 Distribution of Pol II and Pol II-pSer2 on genes induced by p53 activation. a Metagene analysis of Pol II and Pol II-pSer2 occupancy in TSS regions of genes induced by nutlin-3 (*n* = 75; paused genes). The shaded area represents standard deviation (±

s.d.) from the mean distribution (solid line). **b** Box plots comparing ChIP-seq signals in first 100-nt interval downstream of TSS, in the absence or presence of nutlin-3, for Pol II and Pol II-pSer2 at genes induced by nutlin-3, divided into those classified as pause-regulated (Pause index ≥ 2.0, *n* = 75) or non-paused (pause index < 2.0, *n* = 25). **c** Box plots show distribution of Spt5-pSer666 ChIP-seq signals in 100-nt intervals from -100 to +500 bp of TSS (*n* = 75). **d** Box plots show distribution of Spt5-pThr806 ChIP-seq signals in 100-nt intervals from -100 to +500 bp of TSS (*n* = 75). **e** Box plots show enrichment of Spt5 (left), Spt5-pSer666 (middle) and Spt5-pThr806 (right) mean ChIP-seq signals in intervals around the CPS (x = -2.5 kb to 0; y = 0 to +2.5 kb) when cells were treated with nutlin-3 or mock-treated (DMSO) (*n* = 75). Indicated *p*-values were calculated by two-sided Student's *t*-test (**b**, **c**, **d** and **e**). Source data are provided as a Source Data file.

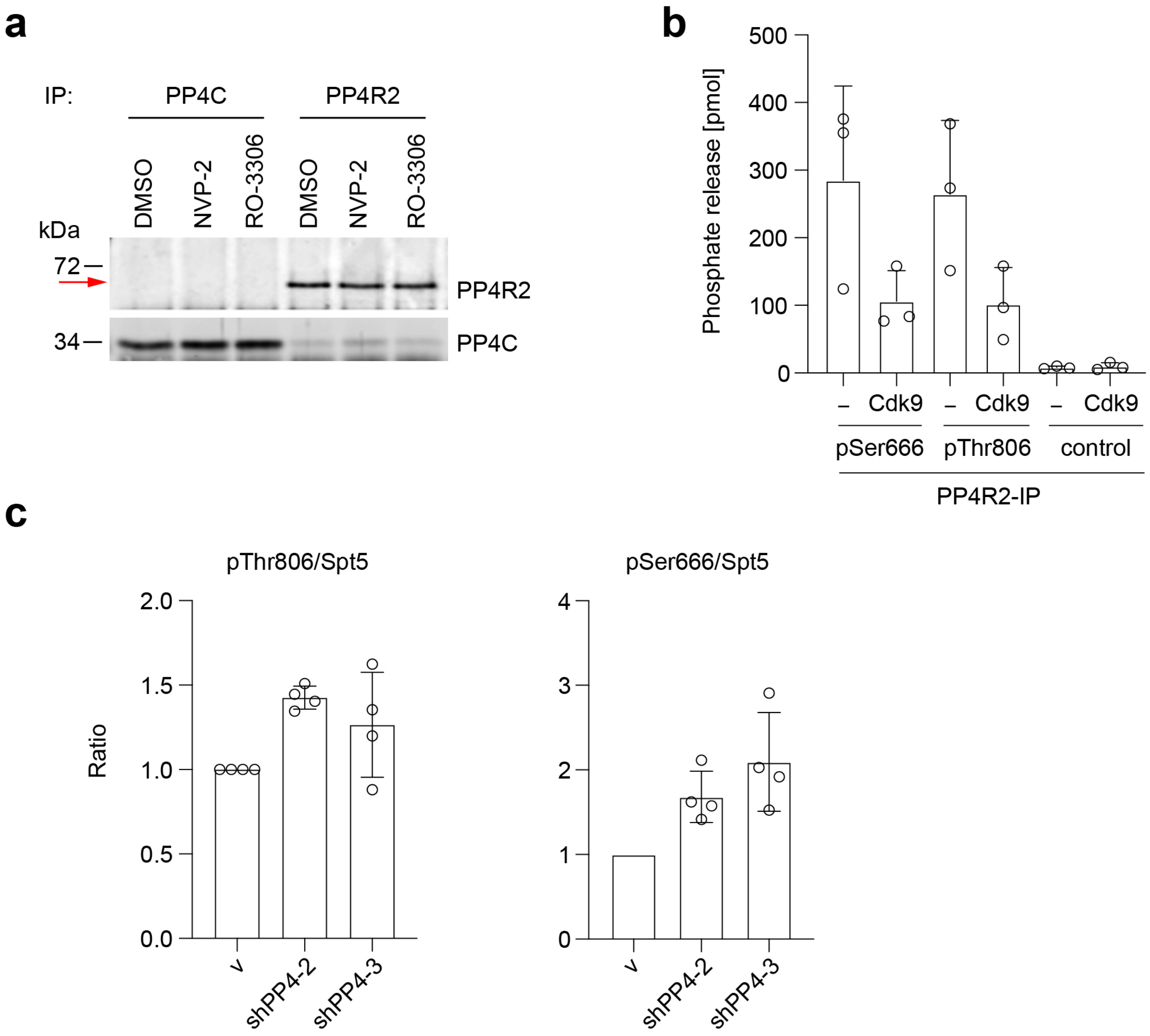

Supplementary Fig. 8 PP4 is a candidate Spt5 phosphatase. a PP4C and PP4R2 were immunoprecipitated from extracts prepared from HCT116 cells mock treated (DMSO) or treated with NVP-2 (250 nM) or RO-3306 (10 μM) for 1 hr. PP4C was co-precipitated with anti-PP4R2 but PP4R2 was not detectable in anti-PP4C immunoprecipitate. **b** Anti-PP4R2 immunoprecipitates were incubated with 5 ng purified, recombinant Cdk9/cyclin T1 and ATP or mock-treated (as indicated), washed and tested for phosphatase activity towards an Spt5-derived phosphopeptide containing pSer666 or pThr806. Error bars indicate + s.d. from three biological replicates (*n* = 3). **c** Quantification of immunoblot results in Fig. 6d. Error bars indicate ± s.d. from 4 biological replicates (*n* = 4). Individual data points from biological replicates are shown in the plots (**b** and **c**). Source data are provided as a Source Data file.

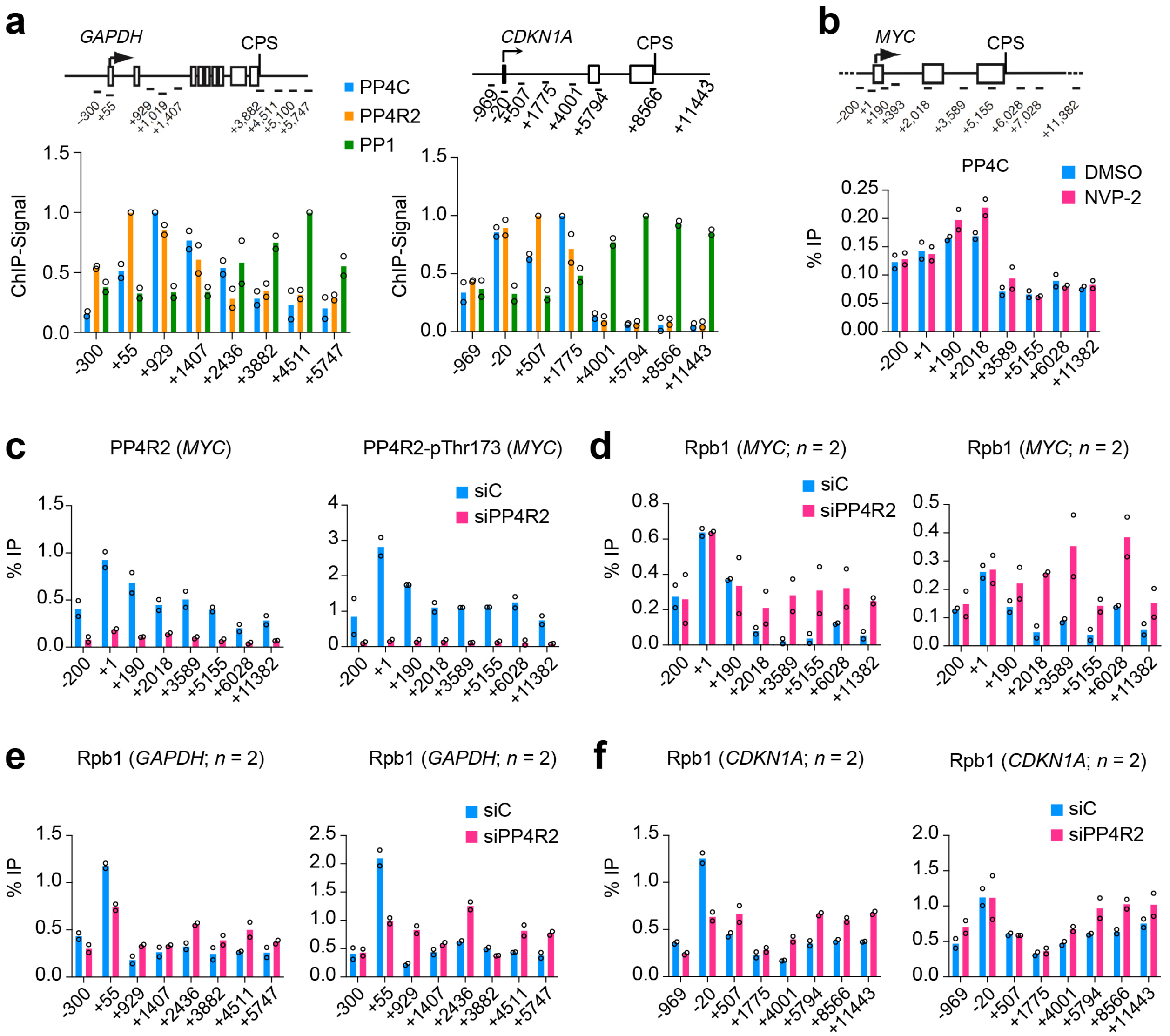

Supplementary Fig. 9 Distinct spatial distributions and substrate specificities of two Cdk9-regulated, phospho-Spt5 phosphatases. a Schematic of the *GAPDH* and *CDKN1A* genes, indicating positions of primer pairs used in ChIP-qPCR analysis (*top*). ChIP-qPCR analysis of PP4C, PP4R2 and PP1γ on the *GAPDH* and *CDKN1A* gene in unperturbed HCT116 cells (*bottom*) reveals reciprocal distribution patterns: PP4 subunits are enriched near the 5' end whereas PP1γ occupancy peaks at the 3' end. **b** Schematic of the *MYC* gene, indicating positions of primer pairs used in ChIP-qPCR analysis (*top*). Inhibition of Cdk9 with NVP-2 (250 nM, 1 hr) does not affect PP4C occupancy on transcribed chromatin. **c** ChIP-qPCR analysis of PP4R2 and PP4R2-pThr173 on the *MYC* gene upon siRNA-mediated depletion of PP4R2 reveals chromatin occupancy of PP4R2 and the Thr173-phosphorylated form is reduced, validating specificity of the anti-pThr173 antibody. **d** ChIP-qPCR analysis of Pol II on the *MYC* gene upon siRNA-mediated depletion of PP4R2. **e** ChIP-qPCR analysis of Pol II on the *GAPDH* gene upon siRNA-mediated depletion of PP4R2. **f** ChIP-qPCR analysis of Pol II on the *CDKN1A* gene upon siRNA-mediated depletion of PP4R2. Individual data points from two independent biological replicates (n = 2) are shown in the plots (**a-f**). Source data are provided as a Source Data file.

Supplementary Table 1. Primers for Site-Directed Mutagenesis

Name	Sequence	Key
SK101	CTGATCCGGGGAGCCATAGGCGCAAAGCCAC	SUPT5H_S666A_F
SK102	GTGGCTTTGCGCCTATGGCTCCCCGGATCAG	SUPT5H_S666A_R
SK103	GCTGATCCGGGGATCCATAGGCGCAAAGCCACC	SUPT5H_S666D_F
SK104	GGTGGCTTTGCGCCTATGGATCCCCGGATCAGC	SUPT5H_S666D_R
PP511	TCATGCAGGGGCGCCTGTGAGCCGTAG	SUPT5H_T806A-F
PP512	CTACGGCTCACAGGCGCCCCTGCATGA	SUPT5H_T806A-R
PP513	CTGCCATCATGCAGGGGATCCTGTGAGCCGTAGTGTG	SUPT5H_T806D-F
PP514	CACACTACGGCTCACAGGATCCCCTGCATGATGGCAG	SUPT5H_T806D-R
PP519	GCCATACATGGGCGCCTGGGAGCCATACA	SUPT5H_T775A-F
PP520	TGTATGGCTCCCAGGCGCCCATGTATGGC	SUPT5H_T775A-R
PP523	TCCTGGAGGGGTGCCTGTGAGCCGTAC	SUPT5H_T791A-F
PP524	GTACGGCTCACAGGCACCCCTCCAGGA	SUPT5H_T791A-R
PP525	CCGTAGTGTGGGGCGCGGCTACCATCC	SUPT5H_T799A-F
PP526	GGATGGTAGCCGCGCCCCACACTACGG	SUPT5H_T799A-R
PP527	TCTGGGCAGGAGCGCGGCTGCCATC	SUPT5H_T814A-F
PP528	GATGGCAGCCGCGCTCCTGCCCAGA	SUPT5H_T814A-R
MS390	CCTTGGAGGCGCTACAGGTCTCGTGGCATTTG	PPP1CC_ T311A_F
MS391	CAAATGCCACGAGACCTGTAGCGCCTCCAAGG	PPP1CC_T311A_R

Fig. 1a

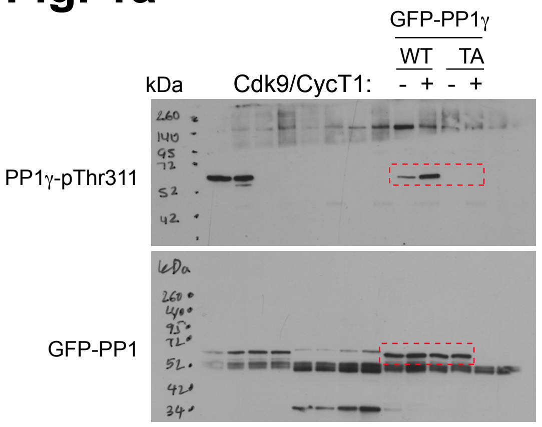

Fig. 1c

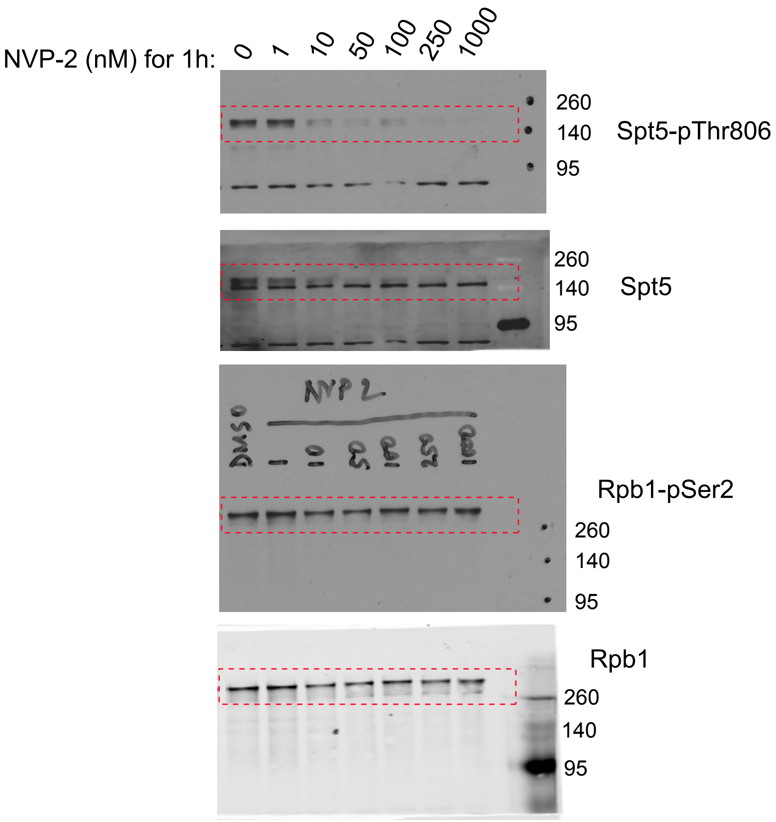

Fig. 1b

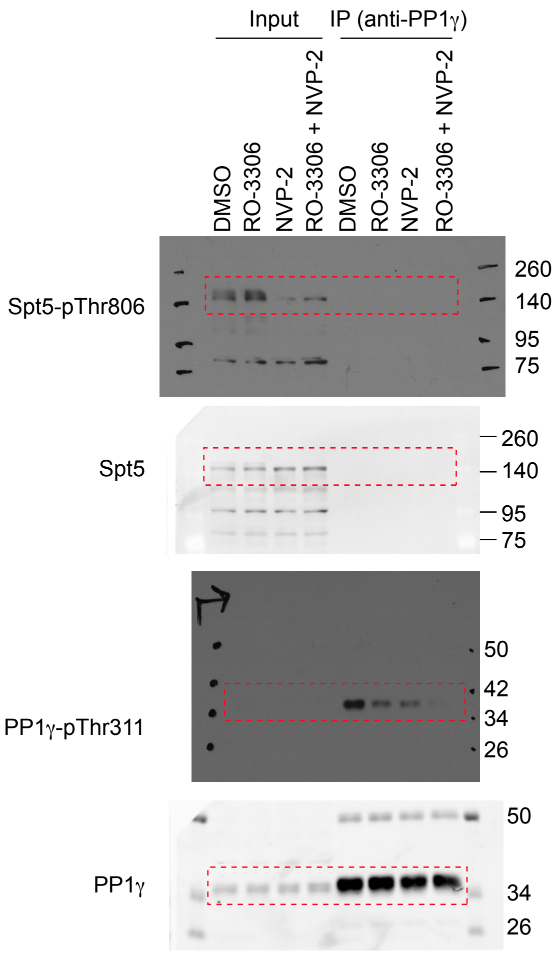

Fig. 1d

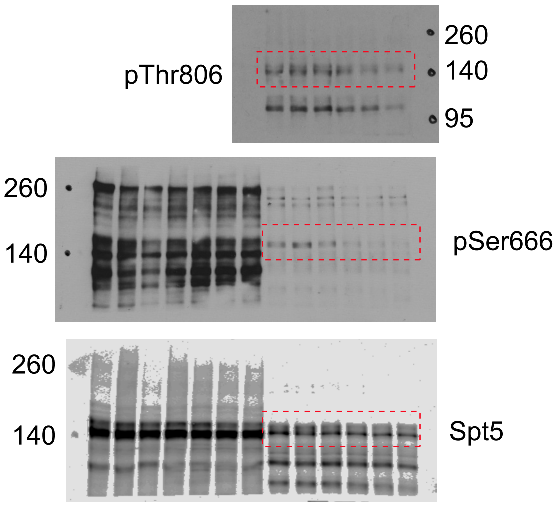

Fig 1f

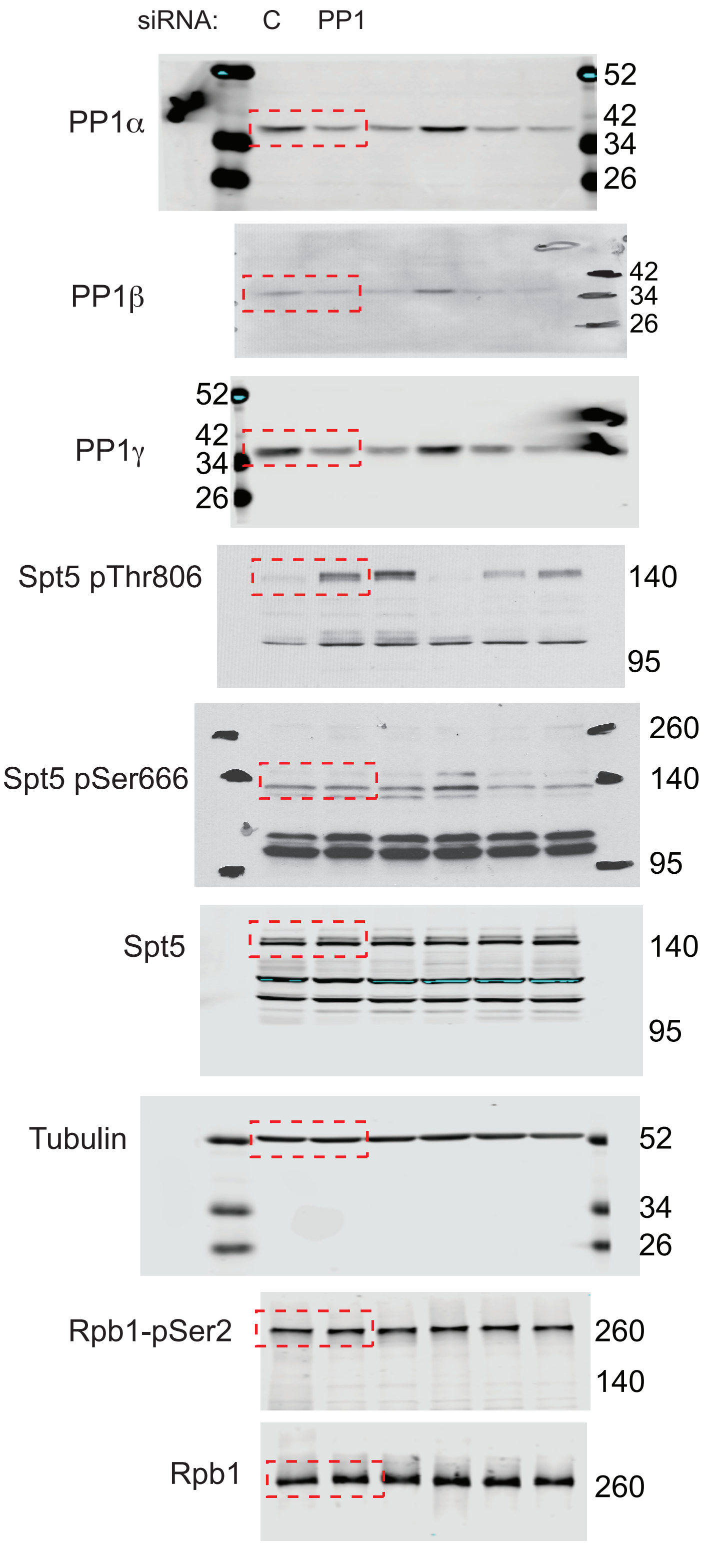
siRNA: C PP1
PP1α
52
42
34
26
PP1β
42
34
26
PP1γ
52
42
34
26
Spt5 pThr806
140
95
Spt5 pSer666
260
140
95
Spt5
140
95
Tubulin
52
34
26
Rpb1-pSer2
260
140
Rpb1
260

Fig. 6a

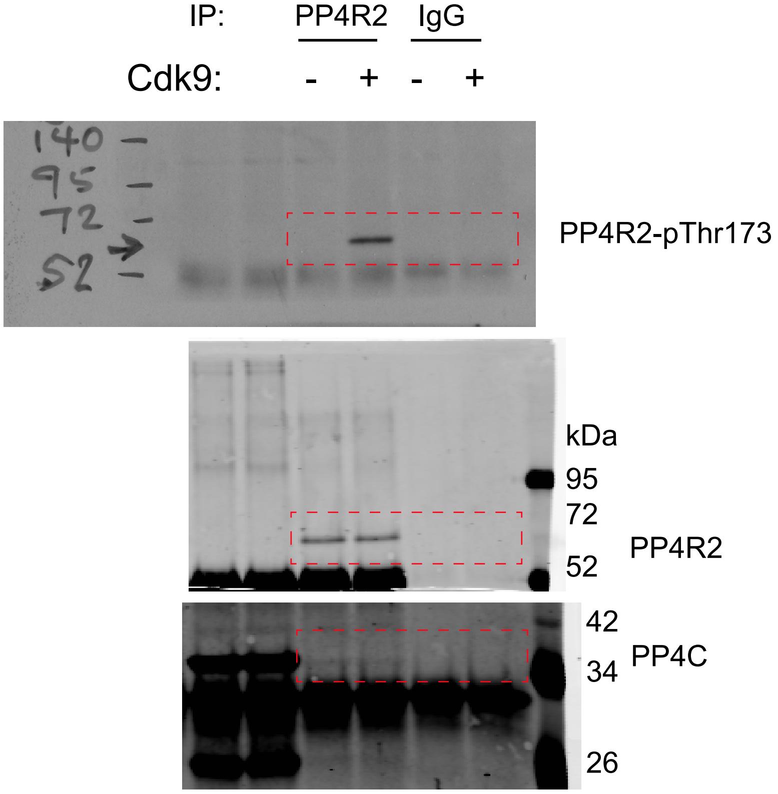
IP:
PP4R2
IgG
Cdk9:
- + - +
140
95
72
52
PP4R2-pThr173
kDa
95
72
52
PP4R2
42
34
26
PP4C

Fig. 6d

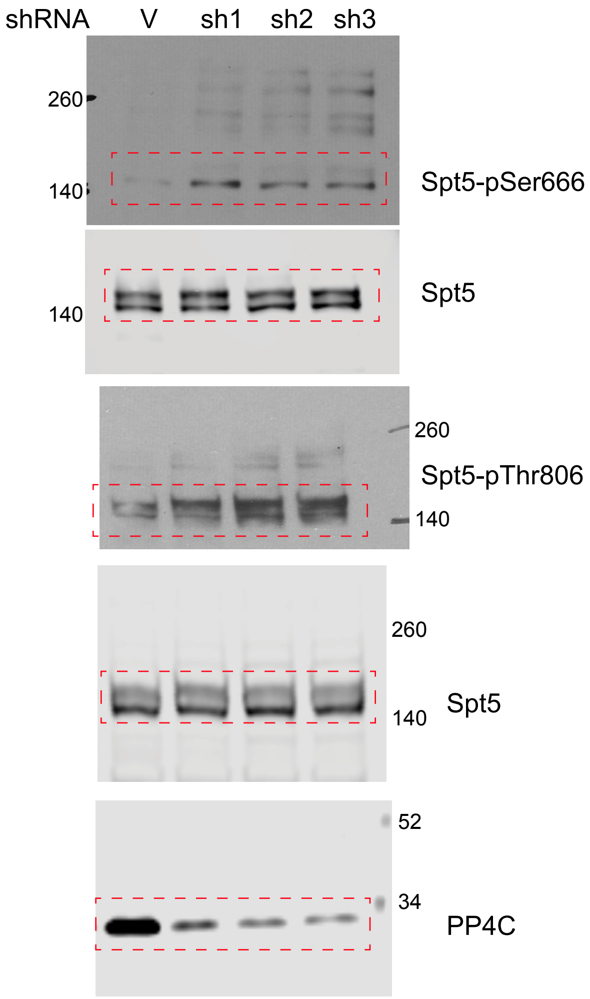
shRNA
V
sh1
sh2
sh3
260
140
Spt5-pSer666
140
Spt5
260
Spt5-pThr806
140
260
140
Spt5
52
34
PP4C

Fig. 7d

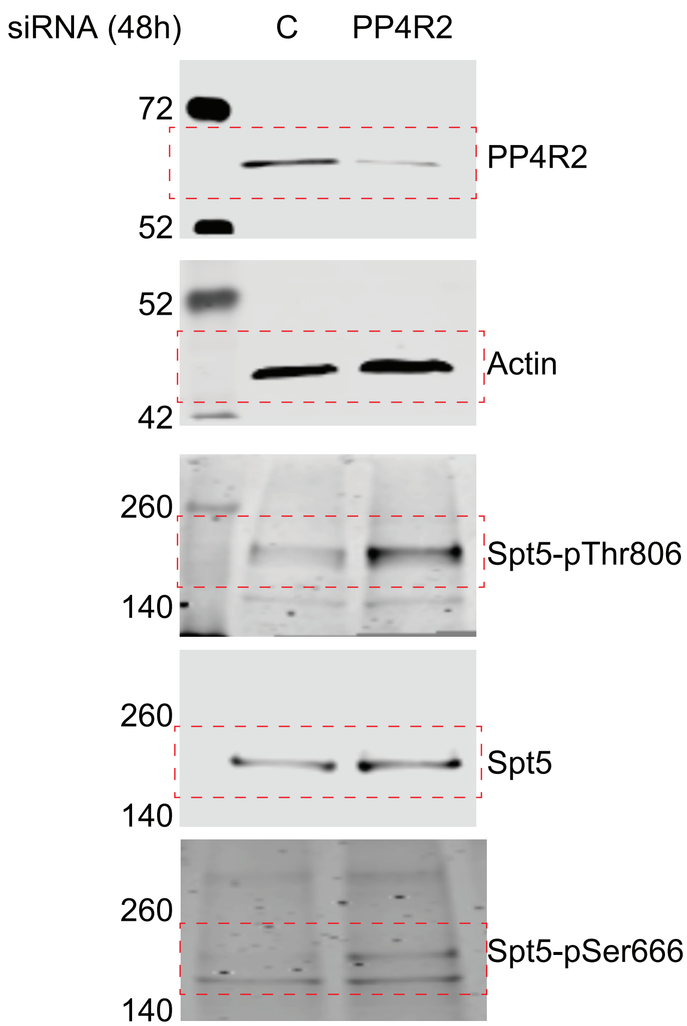
siRNA (48h)
C
PP4R2
72
52
PP4R2
52
42
Actin
260
140
Spt5-pThr806
260
140
Spt5
260
140
Spt5-pSer666

Supp. Fig. 1c

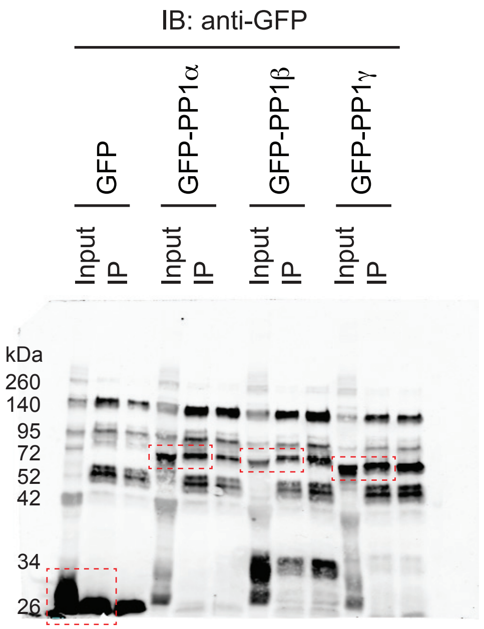

Supp. Fig. 1d

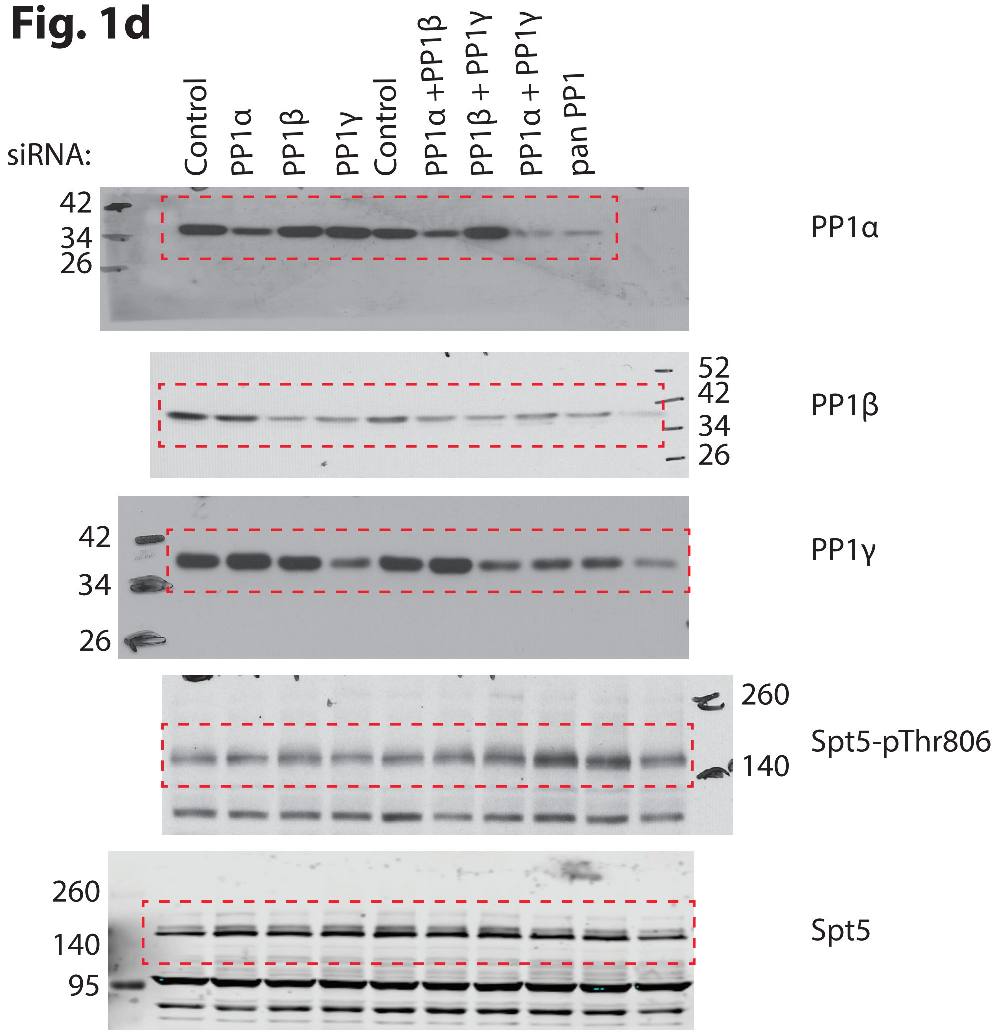

Supp. Fig. 1e

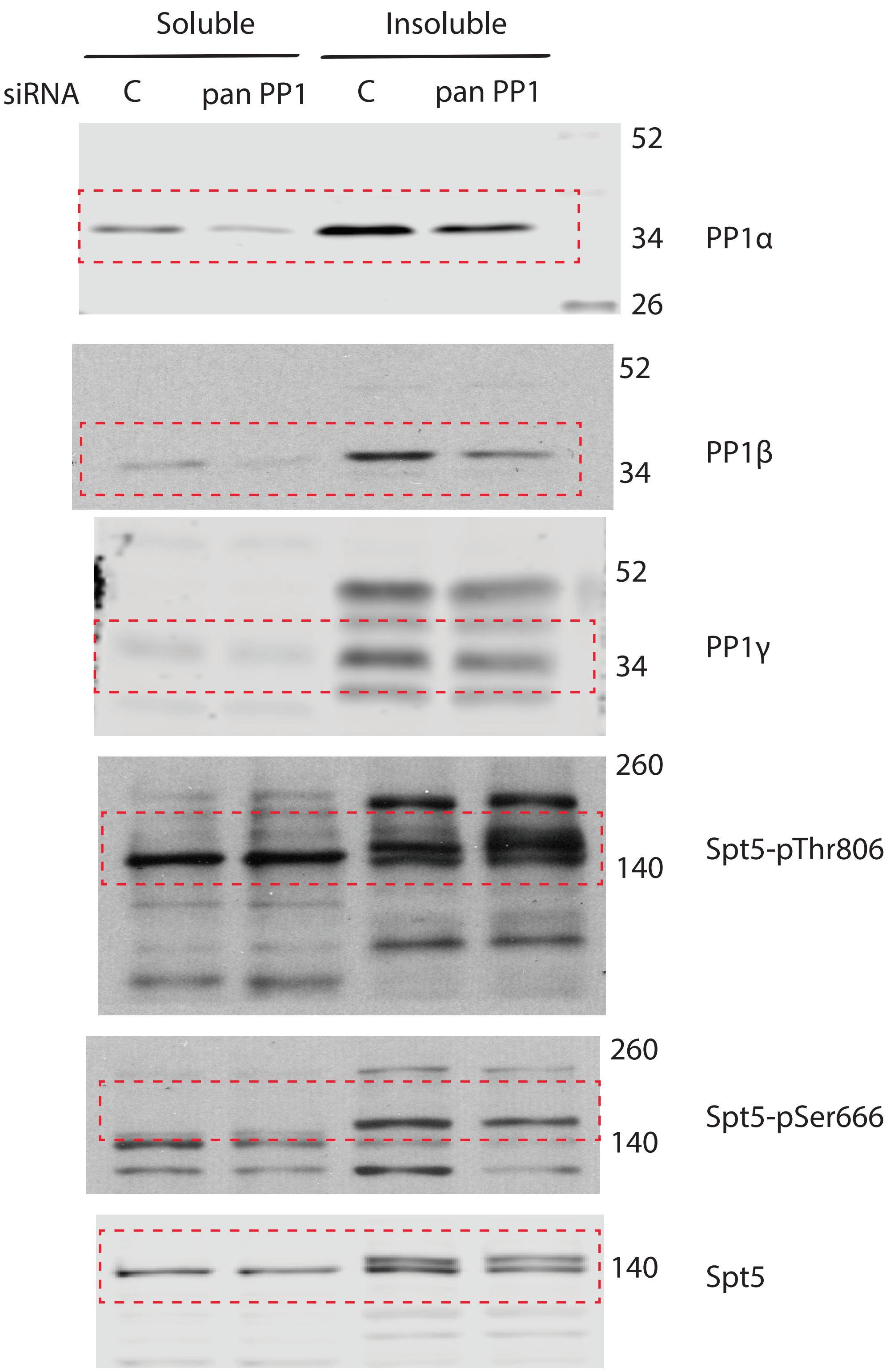
Soluble
Insoluble
siRNA
C
pan PP1
C
pan PP1
52
34
26
PP1α
52
34
PP1β
52
34
PP1γ
260
140
Spt5-pThr806
260
140
Spt5-pSer666
140
Spt5

Supp. Fig. 2a

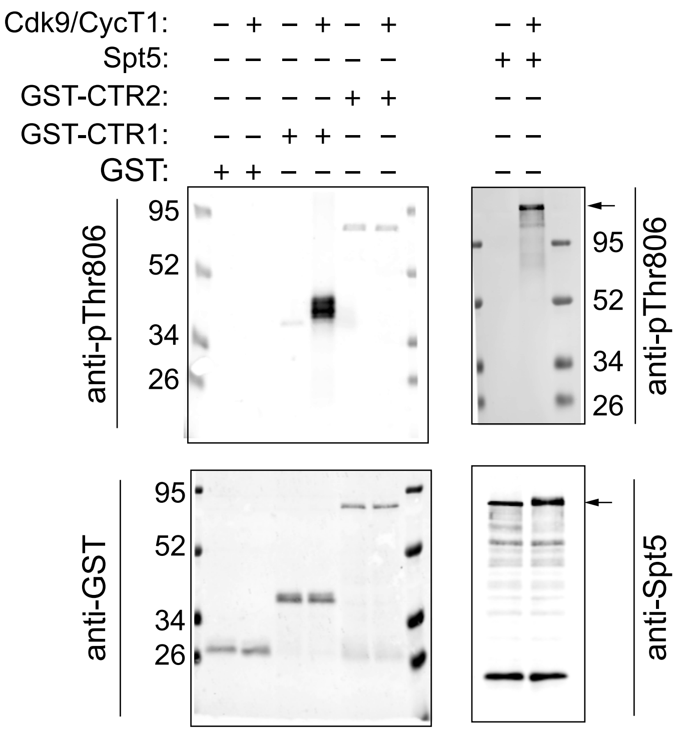

Supp. Fig. 2b

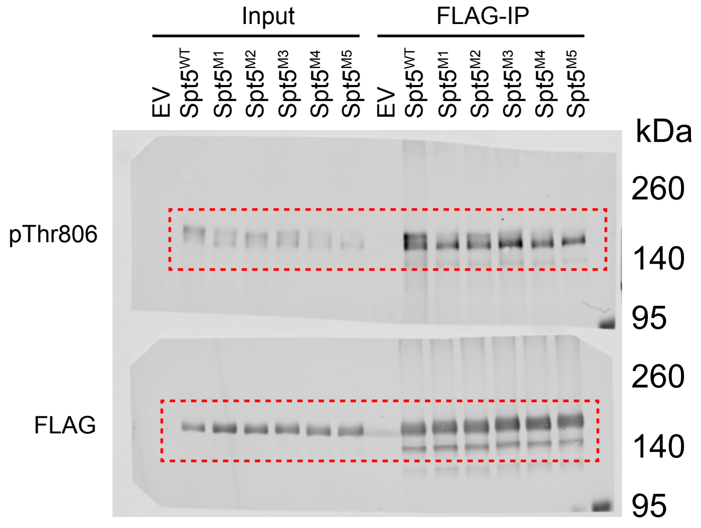

M1: T806A; M2: T775A/T791A/T799A/T814A
M3: T806D; M4: T775A/T806A; M5: T791A/T806A

Supp. Fig. 5a

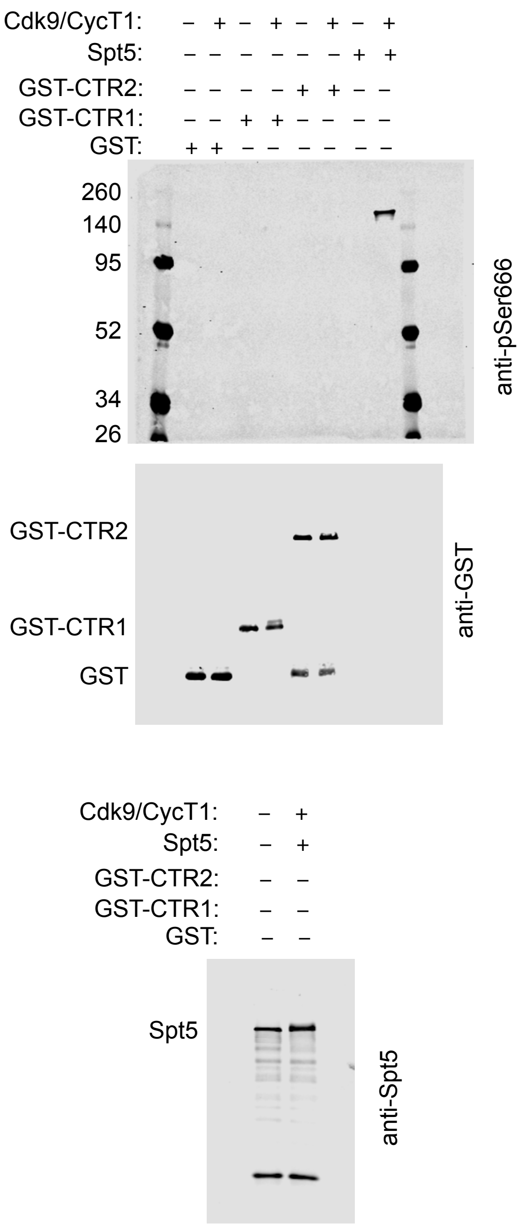

Supp. Fig. 5b

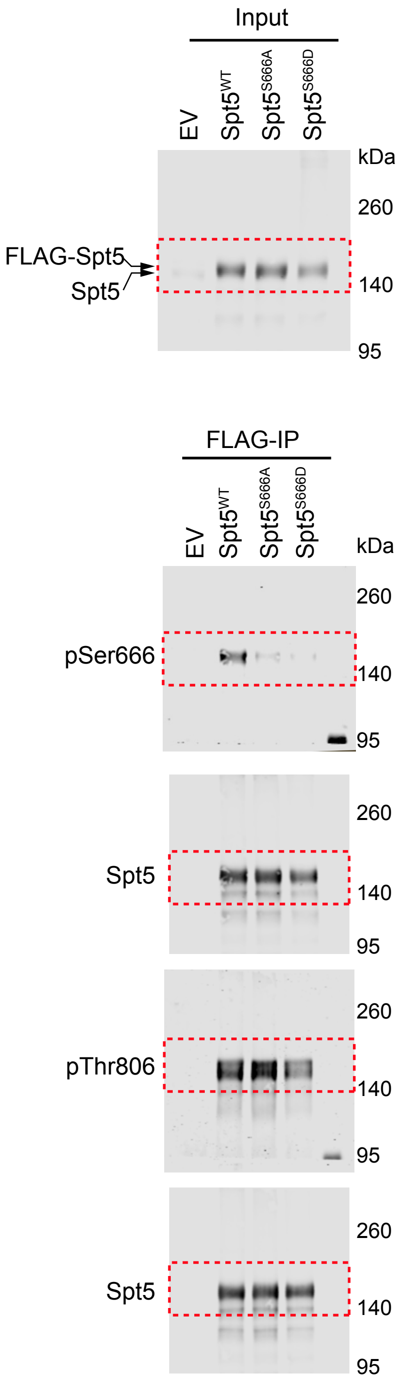

Supp. Fig. 8a

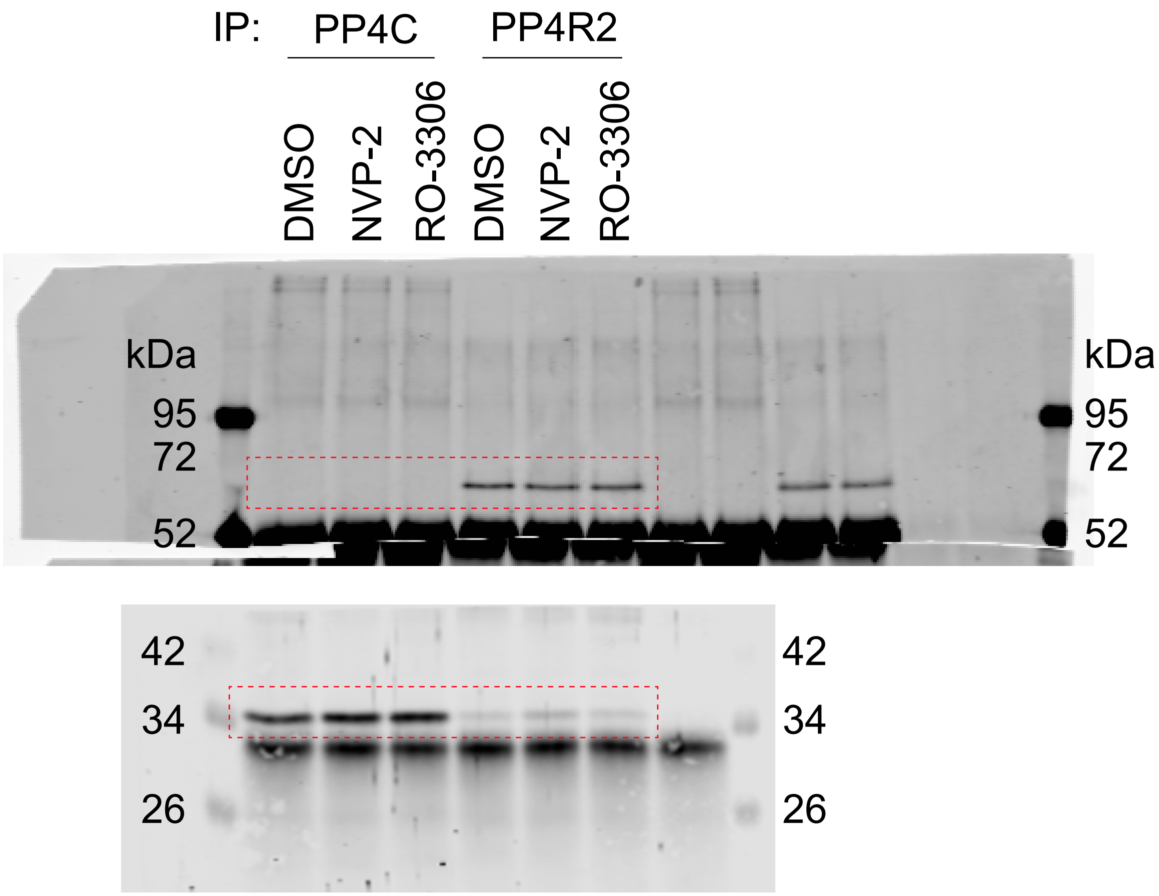
IP:
PP4C
PP4R2
DMSO
NVP-2
RO-3306
DMSO
NVP-2
RO-3306
kDa
95
72
52
kDa
95
72
52
42
34
26
42
34
26
