# Supplementary material for: Distinct Cdk9-phosphatase switches act at the beginning and end of elongation by RNA polymerase II
Source: Nat Commun. 2020 Aug 28;11:4338. doi: 10.1038/s41467-020-18173-6 (PMC7455706; doi:10.1038/s41467-020-18173-6)
Supplement: Supplementary file 3 — Reporting Summary [file 41467_2020_18173_MOESM3_ESM.pdf]

nature research

Corresponding author(s): Robert P. Fisher

Last updated by author(s): 08/05/2020

Reporting Summary

Nature Research wishes to improve the reproducibility of the work that we publish. This form provides structure for consistency and transparency in reporting. For further information on Nature Research policies, see our Editorial Policies and the Editorial Policy Checklist.

Statistics

For all statistical analyses, confirm that the following items are present in the figure legend, table legend, main text, or Methods section.

n/a	Confirmed	
☐	☒	The exact sample size (*n*) for each experimental group/condition, given as a discrete number and unit of measurement
☐	☒	A statement on whether measurements were taken from distinct samples or whether the same sample was measured repeatedly
☐	☒	The statistical test(s) used AND whether they are one- or two-sided<br>*Only common tests should be described solely by name; describe more complex techniques in the Methods section.*
☒	☐	A description of all covariates tested
☒	☐	A description of any assumptions or corrections, such as tests of normality and adjustment for multiple comparisons
☐	☒	A full description of the statistical parameters including central tendency (e.g. means) or other basic estimates (e.g. regression coefficient) AND variation (e.g. standard deviation) or associated estimates of uncertainty (e.g. confidence intervals)
☐	☒	For null hypothesis testing, the test statistic (e.g. *F*, *t*, *r*) with confidence intervals, effect sizes, degrees of freedom and *P* value noted<br>*Give P values as exact values whenever suitable.*
☒	☐	For Bayesian analysis, information on the choice of priors and Markov chain Monte Carlo settings
☒	☐	For hierarchical and complex designs, identification of the appropriate level for tests and full reporting of outcomes
☐	☒	Estimates of effect sizes (e.g. Cohen's *d*, Pearson's *r*), indicating how they were calculated

Our web collection on statistics for biologists contains articles on many of the points above.

Software and code

Policy information about availability of computer code

Data collection

ChIP-qPCR:
ChIP-seq: Sequencing of the ChIP-seq samples was performed on an Illumina NextSeq 500 (Icahn School of Medicine at Mount Sinai).

Data analysis

Immunoblot quantification:
Immunoblots were quantified using Image Studio software in Odyssey CLx Imager (Version 5.2.5) and ImageJ 1.52q.

ChIP-qPCR:
The data were analyzed in Microsoft Excel (Version 14.7.0), Prism 8 (Version 8.4.3). p-values were calculated using two-sided "Student t-test" in Microsoft Excel .

ChIP-seq:
The data were analyzed using Galaxy (https://usegalaxy.org/), an open source and web-based platform. ChIP-seq data analysis softwares and versions in Galaxy are mentioned below--
FastQC Read Quality Reports (Galaxy Version 0.72) and Trimmomatic Flexible Read Trimming Tool (Galaxy Version 0.36.6) to check quality of the sequencing reads and for barcode trimming, respectively. Trimmed sequencing reads were aligned to the human genome (version b37, hg19) using Bowtie2 58 in Galaxy (Galaxy Version 2.3.4.2). Normalization of the aligned reads was done using 'bamCoverage' (Galaxy Version 3.1.2.0.0) by 1) computing and applying scaling factor obtained using aligned sequencing reads of the spike-in reference genome (for spike-in samples) and 2) by computing RPKM (reads per kilobase per million) (for the samples without spike-in control). Aligned sequences of each biological replicate were processed separately to identify enriched binding sites using MACS2 callpeak program 59 (Galaxy Version 2.1.1.20160309.6). The resulting bedgraph files were converted to bigwig using 'Wig/BedGraph-to-bigWig converter' (Galaxy Version 1.1.1), replicates were combined using 'Concatenate datasets' (Galaxy Version 1.0.0). Matrix was computed using 'computeMatrix' (Galaxy v.2.3.6.0) in DeepTools 60 to prepare data for plotting heat maps and/or profiles of given regions. The genome-wide distributions, heat maps and metagene plots were created using 'plotHeatmap' (Galaxy Version 3.1.2.0.1) and 'plotProfile' (Galaxy Version 3.1.2.0.0) tools, respectively.

The phospho-over-total signal ratios (log2-ratio) were calculated using 'bigwigCompare' (Galaxy Version 3.1.2.0.0). To generate principal component analysis (PCA) plots 'plotPCA' (Galaxy Version 3.1.2.0.0) was used.

Box plot:
The box-plots were done using web based software- "www.physics.csbsju.edu/stats/box2.html".

For manuscripts utilizing custom algorithms or software that are central to the research but not yet described in published literature, software must be made available to editors and reviewers. We strongly encourage code deposition in a community repository (e.g. GitHub). See the Nature Research guidelines for submitting code & software for further information.

Data

Policy information about availability of data

All manuscripts must include a data availability statement. This statement should provide the following information, where applicable:

- Accession codes, unique identifiers, or web links for publicly available datasets
- A list of figures that have associated raw data
- A description of any restrictions on data availability

Accession code: Raw and processed ChIP-seq data are deposited in NCBI, and all are available under this accession number GSE138548.
Figure: Figure 2, 4, and 5; Supplementary Figure 2, 3, 5 and 7
Data availability: GSE138548 accession series is released for public.

Field-specific reporting

Please select the one below that is the best fit for your research. If you are not sure, read the appropriate sections before making your selection.

[x] Life sciences [ ] Behavioural & social sciences [ ] Ecological, evolutionary & environmental sciences

For a reference copy of the document with all sections, see nature.com/documents/nr-reporting-summary-flat.pdf

Life sciences study design

All studies must disclose on these points even when the disclosure is negative.

Sample size	Our study does not involve any animals or human samples. All of the experiments were performed with defined laboratory reagents and human cell line(s). Therefore, we did not use any statistical methods to predetermine the sample sizes. Sample sizes were chosen as large as possible while still practically feasible in terms of data collection. Adequate statistics have been applied throughout the manuscript to make sure that the observed effects are significant, given the reported sample size.
Data exclusions	No data were excluded from the analyses.
Replication	Experiments were replicated two to six times with consistent results.<br>ChIP-seq (n = 1-2 biological replicates); ChIP-qPCR (n = 2-4 biological replicates); phosphatase assay (n = 3-6 biological replicates).
Randomization	Our study does not involve any animals or human samples. All of the experiments were performed with defined laboratory reagents and human cell line(s). Therefore, we did not do any randomization in data collection and/or analysis.
Blinding	Our study does not involve any animals or human samples. All of the experiments were performed with defined laboratory reagents and human cell line(s). Therefore, we were not blinded to group allocation during data collection and/or analysis.

Reporting for specific materials, systems and methods

We require information from authors about some types of materials, experimental systems and methods used in many studies. Here, indicate whether each material, system or method listed is relevant to your study. If you are not sure if a list item applies to your research, read the appropriate section before selecting a response.

Materials & experimental systems

n/a	Involved in the study	
[ ]	[x]	Antibodies
[ ]	[x]	Eukaryotic cell lines
[x]	[ ]	Palaeontology and archaeology
[x]	[ ]	Animals and other organisms
[x]	[ ]	Human research participants
[x]	[ ]	Clinical data
[x]	[ ]	Dual use research of concern

Methods

n/a	Involved in the study	
[ ]	[x]	ChIP-seq
[x]	[ ]	Flow cytometry
[x]	[ ]	MRI-based neuroimaging

Antibodies

Antibodies used

Primary antibodies:
The antibodies used were: rabbit anti- Rpb1 (sc-899; Santa Cruz Biotechnology), rabbit anti-Rpb1 (A304-405A & A304-405A, Bethyl Laboratories), rabbit anti-Rpb1 CTD pSer2 (ab5095, Abcam), rabbit anti-Spt5 (A300-868A, Bethyl Laboratories), mouse anti-Spt5 (sc-133217, Santa Cruz Biotechnology), rabbit anti-Spt5-pSer666 and -pThr806 (21st Century Biochemicals), rabbit anti-PP4R2 (A300-838A, Bethyl Laboratories), rabbit anti-PPP4C (A300-835A, Bethyl Laboratories), sheep anti-PP4R2-pThr173 (Division of Signal Transduction Therapy, University of Dundee Scotland), rabbit phospho-PP1α (Thr320) antibody (2581S, Cell Signaling Technology), mouse anti-GFP (sc-9996, Santa Cruz Biotechnology), mouse anti-pan PP1 (sc-7482, Santa Cruz Biotechnology), goat anti-PP1α (sc-6104, Santa Cruz Biotechnology), mouse anti-PP1β (sc-373782, Santa Cruz Biotechnology), rabbit anti-PP1γ (A300-906A, Bethyl Laboratories), goat anti-PP1γ (sc-6108, Santa Cruz Biotechnology), mouse anti-tubulin (T5168, Sigma-Aldrich), rabbit anti-GST (sc-459, Santa Cruz Biotechnology), mouse anti-FLAG® M2 (F3165, Sigma-Aldrich) and rabbit anti-FLAG (2368, Cell Signaling).

Secondary antibodies:
Horseradish Peroxidase (HRP) conjugated donkey anti-rabbit (NA934V, GE Healthcare Life Sciences), sheep anti-mouse (NA9310V, GE Healthcare Life Sciences), donkey anti-sheep (713-035-147, Jackson ImmunoResearch), donkey anti-goat (sc-2020, Santa Cruz Biotechnology).
Alexa Fluor-coupled goat anti-rabbit (A21076, Life Technologies), goat anti-mouse (A11375, Life Technologies), or donkey anti-goat (705-625-147, Jackson ImmunoResearch).

Validation

All antibodies used have been extensively validated by previous publications listed below:

1. Larochelle, S. et al. Cyclin-dependent kinase control of the initiation-to-elongation switch of RNA polymerase II. Nat Struct Mol Biol 19, 1108-1115 (2012). doi: 10.1038/nsmb.2399.
2. Sanso, M. et al. A positive feedback loop links opposing functions of P-TEFb/Cdk9 and histone H2B ubiquitylation to regulate transcript elongation in fission yeast. PLoS Genet 8, e1002822, doi:10.1371/journal.pgen.1002822 (2012).
3. Voss, M. et al. Protein phosphatase 4 is phosphorylated and inactivated by Cdk in response to spindle toxins and interacts with gamma-tubulin. Cell Cycle 12, 2876-2887, doi:10.4161/cc.25919 (2013).
4. Verheyen, T. et al. Genome-wide promoter binding profiling of protein phosphatase-1 and its major nuclear targeting subunits. Nucleic Acids Res., 43(12):5771-84. doi: 10.1093/nar/gkv500 (2015).
5. Qian J, Beullens M, Huang J, De Munter S, Lesage B, Bollen M. Cdk1 orders mitotic events through coordination of a chromosome-associated phosphatase switch. Nat Commun. 2015;6:10215.
6. Dong-Hyun Lee et al. Phosphoproteomic analysis reveals that PP4 dephosphorylates KAP-1 impacting the DNA damage response (2012). EMBO J, May 16;31(10):2403-15. doi: 10.1038/emboj.2012.86.
7. Daniel Hayward et al. Checkpoint signaling and error correction require regulation of the MPS1 T-loop by PP2A-B56 (2019). J. Cell Biol. 2019 Vol. 218 No. 10 3188–3199.

Eukaryotic cell lines

Policy information about cell lines

Cell line source(s)

Colon carcinoma-derived HCT116 cells and human embryonic kidney (HEK293) cells were used in this study. Both cell lines were purchased from ATCC.

Authentication

The cell lines were not authenticated.

Mycoplasma contamination

The cell lines were not tested for mycoplasma contamination.

Commonly misidentified lines
(See ICLAC register)

No commonly misidentified cell lines were used in the study.

ChIP-seq

Data deposition

☒ Confirm that both raw and final processed data have been deposited in a public database such as GEO.

☒ Confirm that you have deposited or provided access to graph files (e.g. BED files) for the called peaks.

Data access links
May remain private before publication.

ChIP-seq data under GEO accession "GSE138548" is now released and publicly available.

Files in database submission

Raw Files:
pp_chipseq_hct116_input_r1_R1.fastq.gz
pp_chipseq_hct116_input_r1_R2.fastq.gz
pp_chipseq_hct116_input_r2_R1.fastq.gz
pp_chipseq_hct116_input_r2_R2.fastq.gz
pp_chipseq_hct116_pol2_r1_R1.fastq.gz

pp_chipseq_hct116_pol2_r1_R2.fastq.gz
pp_chipseq_hct116_pol2_r2_R1.fastq.gz
pp_chipseq_hct116_pol2_r2_R2.fastq.gz
rpf_bb_PolIIpS2_human_ChIP_r1_R1.fastq.gz
rpf_bb_PolIIpS2_human_ChIP_r1_R2.fastq.gz
pp_chipseq_hct116_pol2_pSer2_r2_R1.fastq.gz
pp_chipseq_hct116_pol2_pSer2_r2_R2.fastq.gz
rpf_bb_Spt5total_human_ChIP_r1_R1.fastq.gz
rpf_bb_Spt5total_human_ChIP_r1_R2.fastq.gz
pp_chipseq_hct116_spt5_r2_R1.fastq.gz
pp_chipseq_hct116_spt5_r2_R2.fastq.gz
rpf_bb_Spt5pS666_human_ChIP_r1_R1.fastq.gz
rpf_bb_Spt5pS666_human_ChIP_r1_R2.fastq.gz
rpf_bb_Spt5pT806_human_ChIP_r1_R1.fastq.gz
rpf_bb_Spt5pT806_human_ChIP_r1_R2.fastq.gz
pp_chipseq_hct116_spt5_pt806_r2_R1.fastq.gz
pp_chipseq_hct116_spt5_pt806_r2_R2.fastq.gz
hct116_dmso_input_r2_S1_R1_001.fastq.gz
hct116_dmso_pol2_r2_S5_R1_001.fastq.gz
hct116_dmso_ser2_r2_S6_R1_001.fastq.gz
hct116_dmso_spt5_r2_S2_R1_001.fastq.gz
hct116_dmso_ser666_r2_S3_R1_001.fastq.gz
hct116_dmso_thr806_r2_S4_R1_001.fastq.gz
hct116_nut3_pol2_r2_S10_R1_001.fastq.gz
hct116_nut3_ser2_r2_S11_R1_001.fastq.gz
hct116_nut3_spt5_r2_S7_R1_001.fastq.gz
hct116_nut3_ser666_r2_S8_R1_001.fastq.gz
hct116_nut3_thr806_r2_S9_R1_001.fastq.gz

Processed Files:
bigWig_input_r1.bigwig
bigWig_input_r2.bigwig
bigWig_pol2_r1.bigwig
bigWig_pol2_r2.bigwig
Untreated_Pol2.bigwig
bigWig_Ser2_r1.bigwig
bigWig_Ser2_r2.bigwig
Untreated_Ser2.bigwig
bigWig_Spt5_r1.bigwig
bigWig_Spt5_r2.bigwig
Untreated_Spt5.bigwig
bigWig_SerS666_r1.bigwig
Untreated_Ser666.bigwig
bigWig_Thr806_r1.bigwig
bigWig_Thr806_r2.bigwig
Untreated_Thr806.bigwig
DMSO_input.bigwig
DMSO_Pol2.bigwig
DMSO_pSer2.bigwig
DMSO_Spt5.bigwig
DMSO_pSer666.bigwig
DMSO_pThr806.bigwig
Nut3_Pol2.bigwig
Nut3_pSer2.bigwig
Nut3_Spt5.bigwig
Nut3_pSer666.bigwig
Nut3_pThr806.bigwig

Genome browser session
(e.g. UCSC)

https://genome.ucsc.edu/s/pabitra.parua/hg19_human_parua
https://genome.ucsc.edu/s/pabitra.parua/hg19_human_parua%2Dv2

Methodology

Replicates	Two biological replicates for each sample
Sequencing depth	40 nucleotides in Paired-end sequencing<br>75 nucleotides in Single-end sequencing
Antibodies	Rabbit anti- Rpb1 (sc-899; Santa Cruz Biotechnology)

Rabbit anti-Rpb1 CTD pSer2 (ab5095, Abcam)
Rabbit anti-Spt5 (A300-868A, Bethyl Laboratories)
Rabbit anti-Spt5-pSer666 (21st Century Biochemicals; Sansó et al., Genes Dev. 2016)
Rabbit anti-Spt5-pThr806 (21st Century Biochemicals; Sansó et al., Genes Dev. 2016)

Peak calling parameters

Peak calling was done using MACS2 callpeak (Galaxy Version 2.1.1.20160309.6). Parameters are listed here-
H. sapiens (2.7e9) genome was used as reference genome.
Input files were Single-end or Paired-end BAM.
Band width for picking regions to compute fragment size: 300 bp
Mfold settings: 5 -50
Peak detection based on: q-value
Minimum FDR (q-value) cutoff for peak detection: 0.05
Build model: Shifting model
When set, scale the small sample up to the bigger sample: No
Use fixed background lambda as local lambda for every peak region: No
When set, use a custom scaling ratio of ChIP/control for linear scaling: 1.0
The small nearby region in basepairs to calculate dynamic lambda: 1000
The large nearby region in basepairs to calculate dynamic lambda: 10000
Composite broad regions: No broad regions
Use a more sophisticated signal processing approach to find subpeak summits in each enriched peak region: No
How many duplicate tags at the exact same location are allowed?: 1

Data quality

FastQC Read Quality Reports (Galaxy Version 0.72) was used to analyze and check quality of the raw sequencing data.

Software

Sequencer: Sequencing of the ChIP-seq samples was performed on an Illumina NextSeq 500 (Icahn School of Medicine at Mount Sinai).
Software:
'FastQC Read Quality Reports' (Galaxy Version 0.72) and 'Trimmomatic Flexible Read Trimming Tool' (Galaxy Version 0.36.6) to check quality of the sequencing reads and for barcode trimming, respectively. Trimmed sequencing reads were aligned to the human genome (version b37, hg19) using Bowtie2 58 in Galaxy (Galaxy Version 2.3.4.2). Normalization of the aligned reads was done using 'bamCoverage' (Galaxy Version 3.1.2.0.0) by 1) computing and applying scaling factor obtained using aligned sequencing reads of the spike-in reference genome (for spike-in samples) and 2) by computing RPKM (reads per kilobase per million) (for the samples without spike-in control). Aligned sequences of each biological replicate were processed separately to identify enriched binding sites using MACS2 callpeak program 59 (Galaxy Version 2.1.1.20160309.6). The resulting bedgraph files were converted to bigwig using 'Wig/BedGraph-to-bigWig converter' (Galaxy Version 1.1.1), replicates were combined using 'Concatenate datasets' (Galaxy Version 1.0.0). Matrix was computed using 'computeMatrix' (Galaxy v.2.3.6.0) in DeepTools 60 to prepare data for plotting heat maps and/or profiles of given regions. The genome-wide distributions, heat maps and metagene plots were created using 'plotHeatmap' (Galaxy Version 3.1.2.0.1) and 'plotProfile' (Galaxy Version 3.1.2.0.0) tools, respectively. The phospho-over-total signal ratios (log2-ratio) were calculated using 'bigwigCompare' (Galaxy Version 3.1.2.0.0). To generate principal component analysis (PCA) plots 'plotPCA' (Galaxy Version 3.1.2.0.0) was used.
