# Supplementary material for: Distinct Cdk9-phosphatase switches act at the beginning and end of elongation by RNA polymerase II
Source: Nat Commun. 2020 Aug 28;11:4338. doi: 10.1038/s41467-020-18173-6 (PMC7455706; doi:10.1038/s41467-020-18173-6)
Supplement: Supplementary file 2 — Peer Review File [file 41467_2020_18173_MOESM2_ESM.pdf]

Reviewer #1 (Remarks to the Author):

The work by Parua et all demonstrates a conserved role of distinct phosphatases that work on SPT5 at distinct steps of the transcription cycle. By combining in vitro phosphatase assays with in vivo distribution of transcriptional components following perturbation (by mutation or drug), this work makes a compelling case for further dissection of transcription elongation. As much as genomics is powerful, it is Figure 1 that is the highlight of the manuscript. Overall, it is a well thought out and well controlled piece that merits publication once minor questions are addressed.

Minor points.
55-74. The introduction is hard to follow in places in the last two paragraphs, but especially the one containing lines 55-74. I read it several times and could not quite grasp it. Is there a way to shorten it?

84. Introduce the abbreviation in the Abstract for consistency?

128. Higher sensitivity of Spt5 "CTD" phosphorylation compared to RPB1's is intriguing. Flavopiridol has been shown in several systems to lead to a decrease in Ser2 phosphorylation. This is also stated in lines 194-195. Is this discrepancy due to technicalities (such as timing of treatment and the use of a more specific CDK9 inhibitor) or something else such as functional heterogeneity of Pol II Ser2p populations, differential requirements for CDK9 for Ser2 phosphorylation in different cells, etc?

166-167. The sentence "Reactivity with Spt5…" may be correct technically, but it is not clear what to make of it in Supplementary Fig 2B. It seems to me that the effect of 806 substitution is not only modest (and may be should be quantified), but the IP datapoints do not appear to add useful information.

Supplementary 2d and 2e. These appear to be shown in linear scale. Would it be better to show these in log scale? Secondly, I could not find information (which I may have missed) what signal was quantified for these plots: signal around genes or gene-agnostic data centric peaks?

182. Principal component analysis is highly sensitive to normalization. Normalization is impossible to obtain for ChIP comparing different antibodies. PCA has limited use for samples (such as RNA-seq) that are highly similar and where normalization is straightforward, but it has no use for ChIP-seq. This panel should be removed. Perhaps clustering analysis that is less sensitive to normalization could be used? Overall, this panel does not add much to the manuscript anyway so deleting this will not harm it.

212-214. Heatmaps of different versions of Spt5 are disjointed between figures 3 and 4. I understand that this is done to preserve the flow of the manuscript, but it possible to add a metagene plot overlapping the profiles of wild type and two mutants? It may be worthwhile to make the overlap plot a new 4A and move the orphan heatmap to supplementary, for example.

Lines 221-230. Authors mention transcription induction of CDKN1A. Is transcriptional induction shown somewhere by RT-qPCR? I apologize in advance if I'd missed it.

Lines 233-240. Along the same lines, what is happening to expression and ChIP-seq signal on genes that are not induced by P53 stabilization?

Figure 5B. Metagene plots in 5B are not convincing along the vertical axis. Spike-in based normalization for ChIP-seq is impossible to very difficult with a notable exception of recombinant nucleosomes. A better figure would be to show the same metaplots, but for genes that are not upregulated by Nutlin. These must show less to no increase of light blue signal relatively to genes shown in 5b. This control is also essential.

Figure 7a-c. The gene identity (MYC) is better to be labeled in the figure itself rather than only in a legend to 7B. It takes a moment to decipher that 7C probably shows MYC based on matching primer numbers.

326. Should it be 'marks' (plural)?

380-390. Because these are important statements, these should almost be placed to the beginning of discussion?

392. Delete 'Therefore'.

512. Was Fisher formaldehyde regular or methanol-free that is sold in ampules?

516. Specify the power number for Bioruptor? The high setting for ChIP is considered to be 140 if I recall correctly, but the instrument can go higher.

Data handling. Which samples contained spike-ins?

Data handling. How Sacharomyces spike-ins are helpful when added to immunoprecipitation reactions? The antibodies used will not recognize yeast proteins and will result in unknown and highly variable signal recovery for yeast read among samples that is likely driven by noise.

Reviewer #2 (Remarks to the Author):

Papua et al. address an interesting and important question in gene expression regulation by investigating how SPT5 phosphorylation varies across gene bodies. The authors previously showed in fission yeast that P-TEFb dependent SPT5 phosphorylations were lost at transcription termination sites through competition between the P-TEFb kinase and PP1 phosphatase. Here they have extended these studies to look at the role of various phosphatases during transcription elongation in human cells. The authors probe two different SPT5 phosphorylation sites, Ser666 and Thr806. They find that these phosphorylations are added and lost at different points during transcription. PP1 phosphatases are responsible for removing the phosphorylation on CTR-I at termination sites and PP4 phosphatase removes Ser666 phosphorylation at promoter-proximal sites. This paper is highly important because it shows that in addition to RNA polymerase II, specific sites on SPT5 are phosphorylated in response to its spatial location on a gene body. The study poses an enticing question as to whether other transcription elongation factors are regulated in a similar way. If true, this would provide an additional mechanism to carefully regulate spatial recruitment of additional factors to the elongation machinery. Overall, the manuscript is well written, and the data are clear. If some specific issues are addressed (see below), the reviewer fully supports publication of this important work in Nature Communications.

Specific comments
-Line 49: P-TEFb phosphorylates components of elongation and pausing complexes. The reviewer does not know of additional pausing factors that P-TEFb phosphorylates (NELF and DSIF are already mentioned). The authors should change "the paused complex" to "elongation factors".

-Line 128: The authors should cite the work of Matthias Geyer (2012 Czudnochowski et al., Nature Communications) where it was shown that P-TEFb is not a Ser2 kinase in vitro, specifically because the authors use P-TEFb in vitro in this work.

-Supplementary Fig 1e: Not obvious that chromatin bound Thr806 phosphorylation level is changing in the PP1 depleted sample. It is also not clear that this blot is necessary given the other data in the manuscript.

-Supplementary Fig 2a- Loading controls suggest that there is more GST-CTR1 input than the other peptides (significantly more). The authors comment in the legend that this is due to incomplete stripping of the membrane. This is a really important control, so it is advised the experiment is repeated and the sample is evenly split onto two gels/membranes to show the same amount of sample was indeed used, specifically because some faint bands appear on the phosphoblot for the other CTR constructs. The blots with the full-length protein look good.

-Figure 2B: Are the ChIP plots scaled the same way? Add Y value to plots or state in figure legend that the scaling is the same.

-Figure 2C, 3C: Plot the curves with the same Y axis boundaries to show the extent of differences.

-Figure 3A, 4D, 7A S3A, S4 C, D: Mark termination sites or CPS sites on gene overview. There are probably multiple sites, but it helps to know where transcription may stop on a particular gene.

-Figure 5C: Is it possible to comment on why Nutlin has a greater effect on non-paused genes? The model posited by the authors suggests that Ser666 phosphorylation follows pause release, and it would be naively thought that the effect of Nutlin would be greater on such genes.

-Figure 7e: The authors see increased Pol II in the gene body when PP4R2 is depleted. This can come from enhanced transcription but can also arise from slow Pol II. If at all possible, the authors should investigate Pol II elongation rates in PP4R2 depleted cells using Gro-seq or TT-seq. This would confirm that enhanced Spt5 Ser666 phosphorylation leads to pause release and faster elongation rates.

Minor comment
-Supplementary Figure 3: Flip panel order so they match Figure 3.

RESPONSES TO REVIEWER COMMENTS

Reviewer #1 (Remarks to the Author):

The work by Parua et all demonstrates a conserved role of distinct phosphatases that work on SPT5 at distinct steps of the transcription cycle. By combining in vitro phosphatase assays with in vivo distribution of transcriptional components following perturbation (by mutation or drug), this work makes a compelling case for further dissection of transcription elongation. As much as genomics is powerful, it is Figure 1 that is the highlight of the manuscript. Overall, it is a well thought out and well controlled piece that merits publication once minor questions are addressed.

We thank the reviewer for this positive assessment. We also really like Figure 1!

Minor points.
55-74. The introduction is hard to follow in places in the last two paragraphs, but especially the one containing lines 55-74. I read it several times and could not quite grasp it. Is there a way to shorten it?

We have streamlined and shortened this paragraph in an attempt to make it clearer and easier to digest.

84. Introduce the abbreviation in the Abstract for consistency?

We have now defined KOW motifs in the abstract.

128. Higher sensitivity of Spt5 "CTD" phosphorylation compared to RPB1's is intriguing. Flavopiridol has been shown in several systems to lead to a decrease in Ser2 phosphorylation. This is also stated in lines 194-195. Is this discrepancy due to technicalities (such as timing of treatment and the use of a more specific CDK9 inhibitor) or something else such as functional heterogeneity of Pol II Ser2p populations, differential requirements for CDK9 for Ser2 phosphorylation in different cells, etc?

Basically, the reviewer is correct; the lack of an effect of NVP-2 on pSer2 is due, we believe, to the greater selectivity of this compound, compared to flavopiridol, for Cdk9 (as opposed, for example, to Cdk12 and Cdk13, orthologs of which are mainly responsible for pSer2 in yeast) and the shorter times of treatment we have used. (Longer treatments probably lead to loss of pSer2 indirectly.) As reviewer 2 points out, our data are consistent with careful biochemical analyses of Cdk9 site preferences within the CTD from the Geyer lab, which we now cite.

166-167. The sentence "Reactivity with Spt5…" may be correct technically, but it is not clear what to make of it in Supplementary Fig 2B. It seems to me that the effect of 806 substitution is not only modest (and may be should be quantified), but the IP datapoints do not appear to add useful information.

We have quantified these results, as now shown in revised Supplementary Fig 2b. Single substitution of Thr806 leads to a >50% reduction in antibody reactivity, and slightly greater reductions when combined with other mutations, but other combinations of mutations (leaving Thr806 intact) also diminish this signal. The importance of this information lies simply in its more thorough characterization of an antibody we previously described (Sansó et al., 2016), which is clearly recognizing a repetitive phosphoepitope within CTR1—much like antibodies used to measure Pol II CTD phosphorylations, and in contrast to the site-specific pSer666 antibody described later.

Supplementary 2d and 2e. These appear to be shown in linear scale. Would it be better to show these in log scale? Secondly, I could not find information (which I may have missed) what signal was quantified for these plots: signal around genes or gene-agnostic data centric peaks?

We thank the reviewer for this suggestion; we have changed to a log scale, making the result much clearer. As described in the legend, these are signals around genes.

182. Principal component analysis is highly sensitive to normalization. Normalization is impossible to obtain for ChIP comparing different antibodies. PCA has limited use for samples (such as RNA-seq) that are highly similar and where normalization is straightforward, but it has no use for ChIP-seq. This panel should be removed. Perhaps clustering analysis that is less sensitive to normalization could be used? Overall, this panel does not add much to the manuscript anyway so deleting this will not harm it.

We have taken this suggestion and deleted the principal component analysis; we agree it added little to the manuscript.

212-214. Heatmaps of different versions of Spt5 are disjointed between figures 3 and 4. I understand that this is done to preserve the flow of the manuscript, but it possible to add a metagene plot overlapping the profiles of wild type and two mutants? It may be worthwhile to make the overlap plot a new 4A and move the orphan heatmap to supplementary, for example.

As the reviewer suggested, we now moved the "orphan" heatmap of pSer666 ChIP-seq data to Supplementary Fig 5c. Fig. 4a now shows metagene plots of Spt5, pSer666 and pThr806 overlaid (by necessity with a different y axis for the weaker pSer666 signals). We agree this is a better representation of the important data and thank the reviewer for the suggestion.

Lines 221-230. Authors mention transcription induction of CDKN1A. Is transcriptional induction shown somewhere by RT-qPCR? I apologize in advance if I'd missed it.

*We have added a panel, Supplementary Fig. 6a, showing RT-qPCR measurements of two p53 target genes (*CDKN1A *and* MDM2*) over a time course of nutlin-3 treatment; we chose our time point for ChIP-seq analysis (2 hr) based on these results.*

Lines 233-240. Along the same lines, what is happening to expression and ChIP-seq signal on genes that are not induced by P53 stabilization?

Pol II occupancy is generally reduced by nutlin-3 treatment at non-p53-responsive genes (Reviewer Fig. 1a, b). Expression of GAPDH *mRNA is repressed under these conditions, as shown by RT-qPCR in panel c. The p53-responsive genes are induced, both relative to* GAPDH *(Supplementary Fig. 6a) and in absolute terms (Reviewer Fig. 1d).*

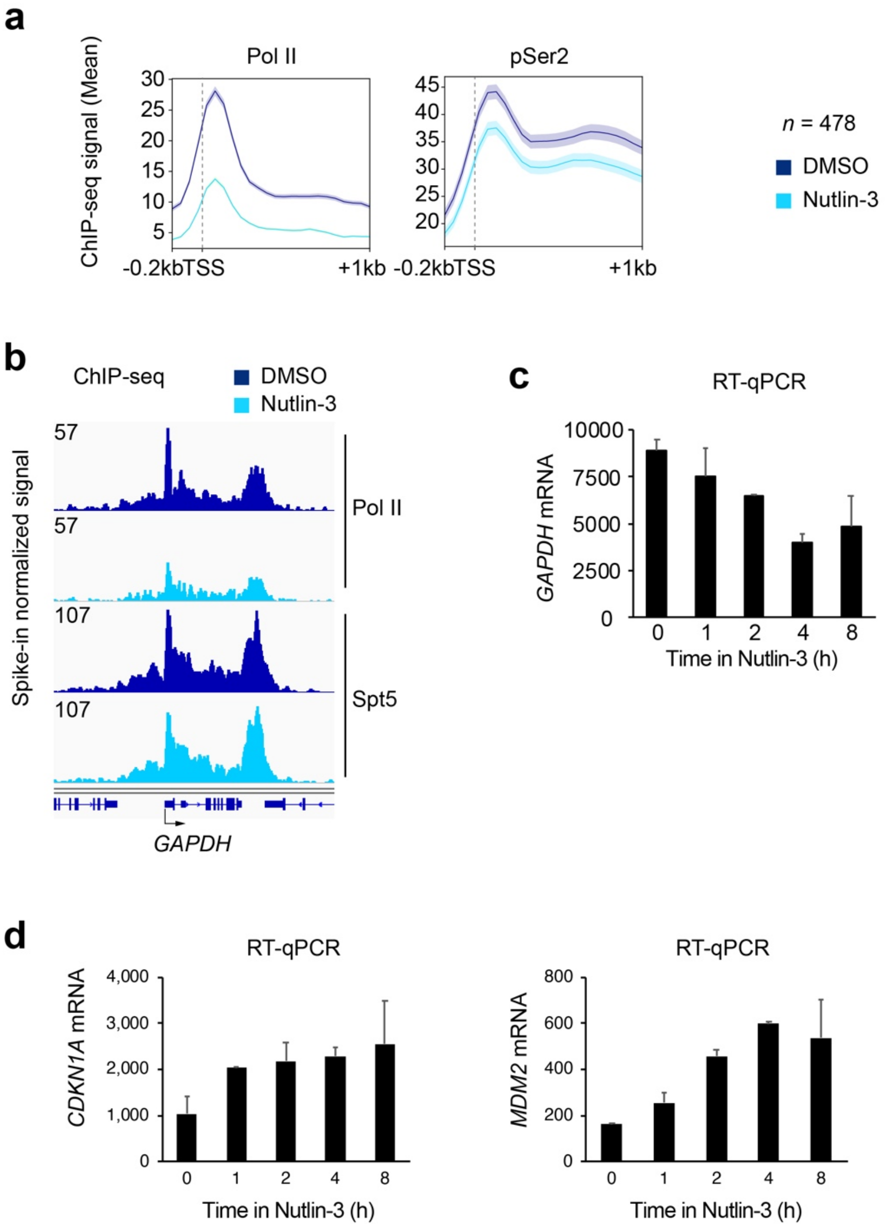

Reviewer Figure 1 Effects of nutlin-3 at non-p53-responsive genes

Figure 5B. Metagene plots in 5B are not convincing along the vertical axis. Spike-in based normalization for ChIP-seq is impossible to very difficult with a notable exception of recombinant nucleosomes. A better figure would be to show the same metaplots, but for genes that are not upregulated by Nutlin. These must show less to no increase of light blue signal relatively to genes shown in 5b. This control is also essential.

We absolutely agree with the reviewer about the usefulness of spike-ins for ChIP-seq analysis (or lack thereof). We have nonetheless included them in more recent experiments because reviewers tend to ask for them (as was the case for a previous submission of this study to a different journal). At that time, we provided side-by-side comparisons of metagene plots generated with or without spike-ins, which we reproduce below (Reviewer Fig. 2) to show that the profiles of the different Spt5 and Pol II modifications are more-or-less the same with either normalization scheme. We agree that the comparison between nutlin-3-induced and non-responsive genes is an important one, and have now included metagene profiles obtained from 478 genes that have high Pol II occupancy but are not induced by p53 stabilization, in Fig. 5c. That analysis reveals little or no change in total Spt5 or pSer666, and a marked reduction of pThr806, indicating that the effects we initially described are indeed specific to p53 induction.

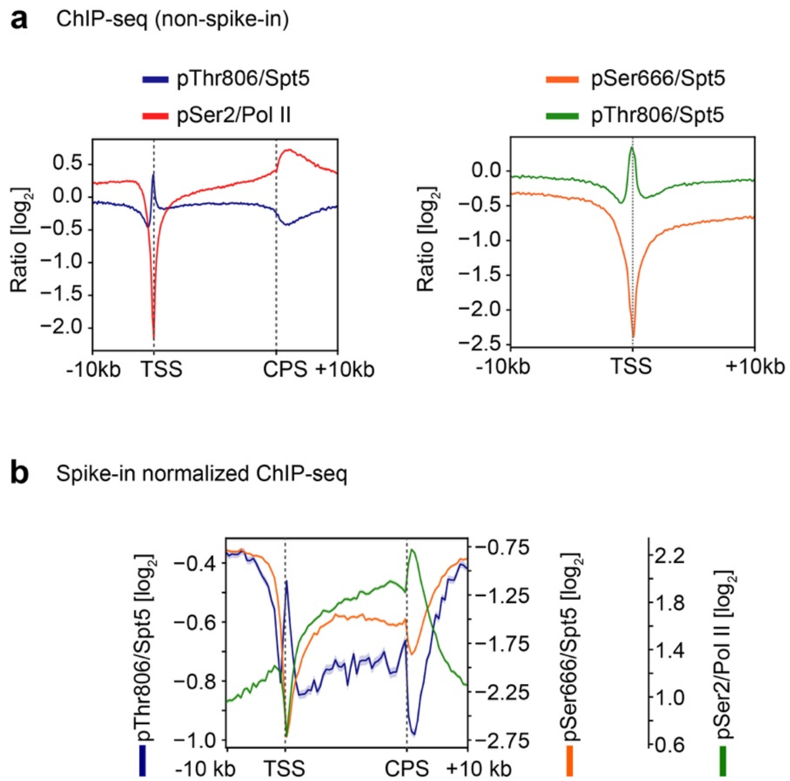

Reviewer Figure 2 Non-spike-in and spike-in-normalized ChIP-seq profiles of Pol II, Spt5 and their respective phosphorylations

Figure 7a-c. The gene identity (MYC) is better to be labeled in the figure itself rather than only in a legend to 7B. It takes a moment to decipher that 7C probably shows MYC based on

matching primer numbers.

We have tried to clarify thus by adding the label "MYC" to the relevant panels.

326. Should it be 'marks' (plural)?

We have changed "mark" to "marks" (although we believe either is correct).

380-390. Because these are important statements, these should almost be placed to the beginning of discussion?

We see the point, but we also like to "finish strong" and this paragraph leads directly into the next (and last) one. If we move one we have to move both, and we're afraid the Discussion would not flow as well. The logic of the current organization is that we start off more narrowly focused on transcriptional machinery and its regulation, and end up putting our results into a more systems-level context (i.e., similarities in phosphoregulation between transcription and cell cycles). We'd be reluctant to lose that progression.

392. Delete 'Therefore'.

Done.

512. Was Fisher formaldehyde regular or methanol-free that is sold in ampules?

We have used both at different times; for the majority of the experiments we used "regular" (for which we now include the catalog number) after ascertaining that there were no differences in the results obtained with either reagent.

516. Specify the power number for Bioruptor? The high setting for ChIP is considered to be 140 if I recall correctly, but the instrument can go higher.

We used an old model of Bioruptor (model/catalog number now listed in Methods), which had no numbers, only "Low," "Medium" and "High" settings. We used "High" as now also indicated.

Data handling. Which samples contained spike-ins?

Spike-ins were used only in the DMSO versus Nutlin-3 experiments (Fig. 5 and Supplementary Fig. 7), as now clearly indicated.

Data handling. How Sacharomyces spike-ins are helpful when added to immunoprecipitation reactions? The antibodies used will not recognize yeast proteins and will result in unknown and highly variable signal recovery for yeast read among samples that is likely driven by noise.

Again, these are all points we made to a previous reviewer as to why spike-in normalization was

not needed to interpret ChIP-seq data properly; we included spike-ins in our nutlin-3 experiment basically to mollify that reviewer. We agree with this reviewer that most antibodies will not recognize yeast proteins reliably or reproducibly (although some, like pSer2, very definitely do), and only compare "like-to-like" (in effect, assuming that the background IP signal will be the same from sample to sample). We have adopted spike-ins to "immunize" ourselves against those reviewers who insist on them, but we get the same results even when we don't use them, i.e., when we normalize our data to irrelevant antibody controls (see Reviewer Fig. 2).

Reviewer #2 (Remarks to the Author):

Papua et al. address an interesting and important question in gene expression regulation by investigating how SPT5 phosphorylation varies across gene bodies. The authors previously showed in fission yeast that P-TEFb dependent SPT5 phosphorylations were lost at transcription termination sites through competition between the P-TEFb kinase and PP1 phosphatase. Here they have extended these studies to look at the role of various phosphatases during transcription elongation in human cells. The authors probe two different SPT5 phosphorylation sites, Ser666 and Thr806. They find that these phosphorylations are added and lost at different points during transcription. PP1 phosphatases are responsible for removing the phosphorylation on CTR-I at termination sites and PP4 phosphatase removes Ser666 phosphorylation at promoter-proximal sites. This paper is highly important because it shows that in addition to RNA polymerase II, specific sites on SPT5 are phosphorylated in response to its
spatial location on a gene body. The study poses an enticing question as to whether other transcription elongation factors are regulated in a similar way. If true, this would provide an additional mechanism to carefully regulate spatial recruitment of additional factors to the elongation machinery. Overall, the manuscript is well written, and the data are clear. If some specific issues are addressed (see below), the reviewer fully supports publication of this important work in Nature Communications.

We thank the reviewer for recognizing the importance of our work and for generally positive comments about the manuscript. Please see below for our responses to the specific concerns raised by this reviewer.

Specific comments
-Line 49: P-TEFb phosphorylates components of elongation and pausing complexes. The reviewer does not know of additional pausing factors that P-TEFb phosphorylates (NELF and DSIF are already mentioned). The authors should change "the paused complex" to "elongation factors".

This passage refers specifically to work from the Cramer lab (Vos et al., refs 18 and 19), who treated reconstituted, paused complexes in vitro with P-TEFb to release the pause. In addition to Pol II, DSIF and NELF, P-TEFb phosphorylated subunits of the PAF complex and Spt6. We see how the original wording might have been confusing, and have revised the problematic sentence to

make clear that we are referring to results of a specific study (which also comes up in the Discussion, lines 324-6), by prefacing it with "In vitro" and putting it in the past tense.

-Line 128: The authors should cite the work of Matthias Geyer (2012 Czudnochowski et al., Nature Communications) where it was shown that P-TEFb is not a Ser2 kinase in vitro, specifically because the authors use P-TEFb in vitro in this work.

We agree; it was an oversight not to cite this work. The Geyer paper is also in good agreement with previous biochemical analyses that indicated no preference of P-TEFb for Ser2. We chose to focus on the previous results that cast doubt on Cdk9's contribution to pSer2 in vivo, but agree the congruence between data in vivo and in vitro should be emphasized (especially given Reviewer 1's comment that the relative insensitivity of pSer2 to NVP-2 is "intriguing").

-Supplementary Fig 1e: Not obvious that chromatin bound Thr806 phosphorylation level is changing in the PP1 depleted sample. It is also not clear that this blot is necessary given the other data in the manuscript.

Although the increase in chromatin-bound pThr806 signal intensity upon PP1 depletion is partly obscured by the concomitant decrease in electrophoretic mobility, that upward shift is itself strong evidence of the gain in phosphorylation. To reinforce this interpretation, we now include quantification of the immunoblot signals in Supplementary Fig. 1e, revealing a nearly 2-fold increase in pThr806:total Spt5, but no change on pSer666. We believe this panel makes the important point that this increase occurs on chromatin (where it matters) and is not occurring at Ser666.

-Supplementary Fig 2a- Loading controls suggest that there is more GST-CTR1 input than the other peptides (significantly more). The authors comment in the legend that this is due to incomplete stripping of the membrane. This is a really important control, so it is advised the experiment is repeated and the sample is evenly split onto two gels/membranes to show the same amount of sample was indeed used, specifically because some faint bands appear on the phosphoblot for the other CTR constructs. The blots with the full-length protein look good.

We agree and have replaced the problematic panel (anti-GST) in Supplementary Figure 2a with one generated from a dedicated, fresh blot.

-Figure 2B: Are the ChIP plots scaled the same way? Add Y value to plots or state in figure legend that the scaling is the same.

For each gene we use the same y-axis scale for each antibody, except anti-pSer666, which gives much lower signals than the rest. We now include y-axis values on each track to clarify this point.

-Figure 2C, 3C: Plot the curves with the same Y axis boundaries to show the extent of differences.

We have now done this. (Note: we believe the reviewer meant Figs. 2c and 4c, not 3c.)

-Figure 3A, 4D, 7A S3A, S4 C, D: Mark termination sites or CPS sites on gene overview. There are probably multiple sites, but it helps to know where transcription may stop on a particular gene.

We have now indicated the most distal annotated CPS on each gene schematic.

-Figure 5C: Is it possible to comment on why Nutlin has a greater effect on non-paused genes? The model posited by the authors suggests that Ser666 phosphorylation follows pause release, and it would be naively thought that the effect of Nutlin would be greater on such genes.

We were also surprised by this. One possible explanation is that the paused genes are where negative elongation factors—including, we would argue, PP4—are most active in opposing P-TEFb action and limiting Spt5 phosphorylation. So at non-paused genes induced by nutlin-3, there is less "resistance" to Cdk9, and pSer666 goes up more dramatically. That is obviously a speculation, which we would not feel comfortable making in print at this point, unless the reviewer and editor feel strongly that this unexpected result needs to be addressed.

-Figure 7e: The authors see increased Pol II in the gene body when PP4R2 is depleted. This can come from enhanced transcription but can also arise from slow Pol II. If at all possible, the authors should investigate Pol II elongation rates in PP4R2 depleted cells using Gro-seq or TT-seq. This would confirm that enhanced Spt5 Ser666 phosphorylation leads to pause release and faster elongation rates.

The reviewer is absolutely correct that decreased rates of elongation might account for increased gene body Pol II occupancy. We tend not to favor this explanation, because the distribution is more-or-less uniform throughout the gene body, with no evidence of 5' accumulation or premature termination. It would also be surprising given that PP4 depletion increases Spt5-CTR1 phosphorylation, which accelerates elongation. We cannot rule it out, however, without a measurement of nascent or ongoing (i.e., run-on) transcription, as the reviewer suggests. We are planning to attempt those analyses (likely by PRO-seq rather than GRO- or TT-seq), but not until we have a more robust, fast-acting method than siRNA or shRNA to reduce PP4 activity. This method development will obviously take time, and some troubleshooting, and is therefore beyond the scope of this, a first report of PP4 influencing Pol II distribution on human genes. In the revised paper we have acknowledged the caveat the reviewer raises (lines 305-7).

Minor comment
-Supplementary Figure 3: Flip panel order so they match Figure 3.

Done.
